# Phase-locking patterns underlying effective communication in exact firing rate models of neural networks

**DOI:** 10.1371/journal.pcbi.1009342

**Published:** 2022-05-18

**Authors:** David Reyner-Parra, Gemma Huguet

**Affiliations:** 1 Departament de Matemàtiques, Universitat Politècnica de Catalunya, Barcelona, Spain; 2 Institut de Matemàtiques de la UPC - Barcelona Tech (IMTech), Barcelona, Spain; 3 Centre de Recerca Matemàtica, Barcelona, Spain; University of Pittsburgh, UNITED STATES

## Abstract

Macroscopic oscillations in the brain have been observed to be involved in many cognitive tasks but their role is not completely understood. One of the suggested functions of the oscillations is to dynamically modulate communication between neural circuits. The Communication Through Coherence (CTC) theory proposes that oscillations reflect rhythmic changes in excitability of the neuronal populations. Thus, populations need to be properly phase-locked so that input volleys arrive at the peaks of excitability of the receiving population to communicate effectively. Here, we present a modeling study to explore synchronization between neuronal circuits connected with unidirectional projections. We consider an Excitatory-Inhibitory (E-I) network of quadratic integrate-and-fire neurons modeling a Pyramidal-Interneuronal Network Gamma (PING) rhythm. The network receives an external periodic input from either one or two sources, simulating the inputs from other oscillating neural groups. We use recently developed mean-field models which provide an exact description of the macroscopic activity of the spiking network. This low-dimensional mean field model allows us to use tools from bifurcation theory to identify the phase-locked states between the input and the target population as a function of the amplitude, frequency and coherence of the inputs. We identify the conditions for optimal phase-locking and effective communication. We find that inputs with high coherence can entrain the network for a wider range of frequencies. Besides, faster oscillatory inputs than the intrinsic network gamma cycle show more effective communication than inputs with similar frequency. Our analysis further shows that the entrainment of the network by inputs with higher frequency is more robust to distractors, thus giving them an advantage to entrain the network and communicate effectively. Finally, we show that pulsatile inputs can switch between attended inputs in selective attention.

## Author summary

Oscillations are ubiquitous in the brain and are involved in several cognitive tasks but their role is not completely understood. The Communication Through Coherence theory proposes that background oscillations in the brain regulate the information flow between neural populations. The oscillators that are properly phase-locked so that inputs arrive at the peaks of excitability of the receiving population communicate effectively. In this paper, we study the emerging phase-locking patterns of a network generating PING oscillations under external periodic forcing, simulating the oscillatory input from other neural groups. We identify the conditions for optimal phase-locking and effective communication. Namely, we find that inputs with higher frequency and coherence have an adavantage to entrain the network and we quantify how robust are to distractors. Furthermore, we show how selective attention can be implemented by means of phase locking and we show that pulsatile inputs can switch between attended inputs.

## 1 Introduction

Macroscopic oscillations are widely observed in the brain and they span a temporal scale that ranges from a few to a hundred hertz [1]. There are several studies that associate these rhythms to different cognitive tasks, but its physiological origin and functional role is not completely understood and constitutes an active area of research [2–5].

Amongst these rhythms, oscillations in the gamma frequency band (30–100 Hz) have been reported in many cortical areas and across different species in a variety of tasks, including attention and memory [5, 6]. Investigations on the mechanisms generating gamma-band oscillations have identified a key role for interneurons [4, 7], which are responsible for generating synaptic inhibitory activity that periodically modulates the excitability of the neurons. These rhythmic changes in the neuronal excitability have been hypothesized to regulate the information flow between distant brain areas in a flexible way. Thus, Communication Through Coherence (CTC) theory [8, 9] proposes that an effective transmission of information between two oscillating neuronal groups occurs when the pre-synaptic input of the sending population reaches systematically the post-synaptic (receiving) population at its maximum phase of excitability (when inhibition has cleared out or is at its lowest value), producing an amplification of the firing rate of the post-synaptic group. The oscillators must be therefore properly phase-locked to accomplish effective communication. In this context, synchrony provides a dynamic mechanism to modulate the information flow without modifying anatomical connections, resulting in functional connectivity [10, 11]. Out of this theory, it emerges a solid hypothesis to explain how selective communication arises in the brain, that is, the ability of a receiving neuronal group to respond selectively to different input streams [9].

A growing number of experimental studies have tested some CTC predictions [9]. In particular, some studies have linked the phase of the inhibitory population with the modulation of the input gain of the target population [12]. Also, effective connectivity has been linked to the phase relation between gamma rhythms at the pre and post-synaptic neuronal groups [13]. On the other hand, different studies support that visual and motor selective attention can be implemented through the control of the phase and synchronization between populations [14–16].

There are also several modeling studies approaching different aspects of the CTC hypothesis. These studies are based either on computational simulations of spiking networks [17–21] or models that admit an analytically more tractable approach such as single neuron models [22, 23], or firing rate models [10, 24]. There are also studies that compare different modeling approaches [10, 25], but when both spiking and mean firing rate models are considered [10], the latter are not an exact derivation of the former. In the last years, the development of a new generation of neural mass models that provide an exact description (in the limit of an infinitely large number of neurons) of the macroscopic quantities of a spiking network [26–28] has opened the possibility to test CTC hypothesis in realistic models, while simplifying the mathematical analysis. Compared to the classical firing rate models, this description is advantageous given that it allows for a direct comparison with the spiking network.

In this study, we consider a spiking network of excitatory and inhibitory cells (E-I network), whose macroscopic activity in terms of mean firing rate and membrane potential can be described exactly by means of a low-dimensional mean-field model [26, 29]. The low-dimensional mean field model permits mathematical analysis using tools from dynamical systems, and thereby prediction and insight into the relevant parameters that characterize the firing properties of the network. These predictions can then be tested in simulations of the full spiking network. This approach allows us to go beyond our results on CTC obtained in an earlier study for the Wilson-Cowan model [24] by considering more realistic networks. In particular, the model shows oscillatory behavior for a larger set of parameters, including the absence of recurrent coupling, and captures transient spike synchronization phenomena within the network.

Specifically, we consider a mutually interconnected network of Excitatory and Inhibitory neurons (E-I network), accounting for macroscopic oscillations via the classical PING (Pyramidal Interneuronal Network Gamma) mechanism [4, 7, 30]. These oscillations describe rhythmic changes in the excitability of the E-cells. We apply to this network an external oscillatory input, modeling the input from the pre-synaptic population, for which we modulate the amplitude, the frequency and coherence (that is, how concentrated input volleys are to particular phases of the oscillation cycle).

The present study requires first to examine how a neural oscillator responds to incoming perturbations and the mathematical tool to characterize it is the *phase response curve* (PRC) [31, 32]. The weak coupling assumption enables us to reduce the dynamics close to the oscillator to a single equation, known as the *phase equation* [33]. Since the perturbation is periodic, we define the so-called *stroboscopic map* for the phase equation, whose fixed and periodic points correspond to different phase-locking states between the input and the target network. A bifurcation analysis of the stroboscopic map determines the Arnold tongues, that is, the regions in the parameter space of amplitude and frequency of the periodic forcing, where the different phase-locked states are located. We find that the pre-synaptic input can entrain the post-synaptic network at different frequencies, and that the range of frequencies is larger for high coherent inputs.

We explore in detail the solutions that emerge in the phase-locking regions to quantify the communication efficiency in the CTC context, that is, we measure how much the target network (receiver) detects the changes in the amplitude of the pre-synaptic inputs (emitter) by means of the firing rate and spike synchronization of the E-cells. Interestingly, we observe that phase-locking is not enough to ensure communication. Indeed, we find that when the input frequency is higher than the natural gamma cycle of the receiver, increasing the strength of the pre-synaptic input produces a significant increase of the synchronization of the E-cells (described by the amplification of the oscillations of the firing rate of the E-cells), but not if the input frequency is similar to the natural gamma cycle. So, communication is more effective in the former case, because the input strength is better transferred and detected by the firing rate synchronization within the target network. Moreover, we explore the effects of a disruptor onto the phase-locking and we observe that those inputs with higher frequency are more robust to disturbances, irrespective of its coherence. As a result, the frequency relationship is crucial to preserve communication in the presence of distracting inputs. Finally, we give particular attention to show that a pulse delivered at the appropriate phase can drive selective attention between two similar oscillatory input streams.

## 2 Results

### 2.1 Mathematical setting for CTC

In this Section we describe the mathematical model for a canonical cortical neural network with intrinsic oscillatory activity in the gamma range. This model will be used to study the effects of an oscillatory input (modeling the input from a population coding for a particular stimulus) onto the network activity. In particular, we will explore phase-locking properties between emitting and receiving populations, and its implications for Communication Through Coherence (CTC) theory, that is, how changes in some input parameters are detected at the output.

Several studies on gamma-frequency oscillations have provided the fundamental neurological requirements to generate them: either from the interplay of excitatory (E) and inhibitory (I) populations or from a single inhibitory population with self-feedback. These two processes are referred to as PING (Pyramidal Interneuron Network Gamma) and ING (Interneuron Network Gamma) mechanisms, respectively, and represent the classical models to generate and explain gamma oscillations [4, 7]. In both mechanisms, inhibition plays a strong role. In this paper we focus on the PING mechanism.

**Fig 1:**
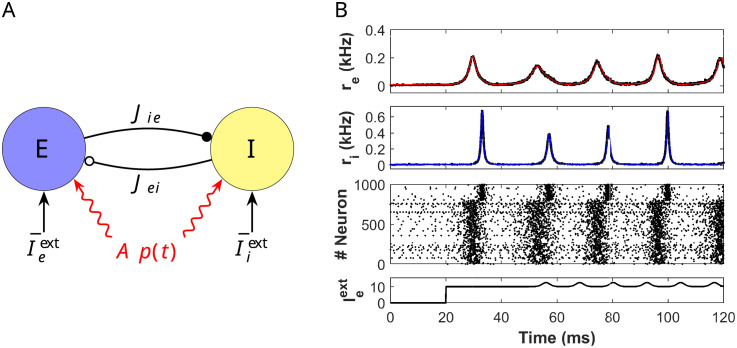
E-I cortical network and comparison between full network and reduced system. (A) Schematic representation of a cortical neural network consisting of excitatory (E) and inhibitory (I) cells. Excitatory (resp. inhibitory) synapses are depicted by arrows with a black-filled (resp. empty) circle pointing at the receiving population. The parameter *J*_*ab*_, *a*, *b* ∈ {*e*, *i*} is the connectivity strength of the *b* → *a* synapse. Besides synaptic input, each population also receives an external tonic current ${\bar{I}}_{a}^{\text{ext}}$, *a* ∈ {*e*, *i*}. Additionally, both populations receive a periodic input *Ap*(*t*) (red curve), modeling the input from an oscillating neuronal population. (B) From bottom to top: Time evolution of the external input current $I_{e}^{\text{ext}}\left(t\right)$ onto the E-cells. $I_{e}^{\text{ext}}\left(t\right)=0$ for 0 < *t* < 20, $I_{e}^{\text{ext}}\left(t\right)=10$, for 20 < *t* < 50 and $I_{e}^{\text{ext}}\left(t\right)=10+0.8p\left(t\right)$, with *p*(*t*) defined in (15) with *κ* = 2, *T* = T*/2 and T* = 24.234 ms. Raster plot of 1000 randomly selected neurons (the first 800 neurons are excitatory and the last 200 inhibitory). Time evolution of the macroscopic quantities *r*_*e*_ and *r*_*i*_ obtained from simulations of the mean-field model (1)-(2) (red and blue curves, respectively) and the averaged firing rate activity of the full spiking network (black). Notice that curves lie one on top of the other, showing perfect agreement. In the latter case, the mean firing rate has been computed by averaging the number of spikes in a time window of size *δt* = 8 ⋅ 10^−2^. Parameters: *N*_*e*_ = *N*_*i*_ = 5000, ${\bar{I}}_{i}^{\text{ext}}=0$ and the rest of the parameters as in (6).

To implement the PING mechanism we consider two populations of neurons, one of excitatory (pyramidal) cells and the other one of inhibitory (interneuron) cells, interconnected synaptically. With sufficiently strong and persistent external excitatory drive to the pyramidal neurons, the E-cells activate and recruit the I-cells which, in turn, send a reciprocal inhibitory feedback onto the E-cells. The inhibition causes the E-population to be less receptive to the external drive until its effect decays, starting again the E-I cycle. This mechanism generates self-sustained macroscopic oscillatory activity in the gamma range (see Fig 1).

To model the E-I network we consider a neural mass model that was recently derived for networks of spiking neurons [26, 29]. It provides an exact description (in the thermodynamic limit) of the macroscopic quantities of a spiking network of quadratic integrate-and-fire neurons, namely, the mean firing rate *r* and the mean membrane potential *V* (see Methods for more details).

The model is given by a set of differential equations for the E-population,

$$\begin{array}{c} \begin{array}{cc} \;\tau_{e}{\dot{r}}_{e} & =\frac{\Delta_{e}}{\pi \tau_{e}}+2r_{e}V_{e}\;,\\ \;\tau_{e}{\dot{V}}_{e} & =V_{e}^{2}+\eta_{e}+I_{e}-{\left(\tau_{e}\pi r_{e}\right)}^{2}\;,\\ \;\tau_{s_{e}}{\dot{S}}_{ee} & =-S_{ee}+J_{ee}r_{e}\;,\\ \;\tau_{s_{i}}{\dot{S}}_{ei} & =-S_{ei}+J_{ei}r_{i}\;, \end{array} \end{array}$$ (1)

and another identical set for the I-population,

$$\begin{array}{c} \begin{array}{cc} \;\tau_{i}{\dot{r}}_{i} & =\frac{\Delta_{i}}{\pi \tau_{i}}+2r_{i}V_{i}\;,\\ \;\tau_{i}{\dot{V}}_{i} & =V_{i}^{2}+\eta_{i}+I_{i}-{\left(\tau_{i}\pi r_{i}\right)}^{2}\;,\\ \;\tau_{s_{e}}{\dot{S}}_{ie} & =-S_{ie}+J_{ie}r_{e}\;,\\ \;\tau_{s_{i}}{\dot{S}}_{ii} & =-S_{ii}+J_{ii}r_{i}\;. \end{array} \end{array}$$ (2)

Here, *V*_*a*_ and *r*_*a*_ represent the mean voltage and mean firing rate of the population *a* ∈ {*e*, *i*}. The parameters *η*_*a*_ and Δ_*a*_ are, respectively, the center and width of the static distribution of inputs to the individual spiking neurons, which is considered to be Lorentzian. The time constant *τ*_*a*_ is the membrane time constant of the individual neurons in population *a*. The variable *S*_*ab*_ models the synaptic current from the pre-synaptic population *b* to the post-synaptic population *a*, and *J*_*ab*_ is the strength of the corresponding synaptic connection. The parameter $\tau_{s_{e}}$ corresponds to the decay time associated to fast excitatory (AMPA) synapses, and $\tau_{s_{i}}$ to the time constant of the fast inhibitory (GABA_*A*_) synapses.

The total input current to the excitatory population is given by

$$\begin{array}{c} I_{e}=I_{e}^{\text{ext}}+\tau_{e}S_{ee}-\tau_{e}S_{ei}\;, \end{array}$$ (3)

and to the inhibitory one, by

$$\begin{array}{c} I_{i}=I_{i}^{\text{ext}}+\tau_{i}S_{ie}-\tau_{i}S_{ii}\;. \end{array}$$ (4)

Notice that the terms *I*_*e*_ and *I*_*i*_ above provide the coupling between systems (1) and (2). The terms $I_{e}^{ext}$ and $I_{i}^{ext}$ represent the external input drive.

In this study, we are going to consider that the input current to the E and I-cells is of the form

$$\begin{array}{c} I_{e,i}^{ext}={\bar{I}}_{e,i}^{ext}+\tau_{e,i}g\left(t\right), \end{array}$$ (5)

where ${\bar{I}}_{e,i}^{ext}$ is a tonic current and *g*(*t*) = *Ap*(*t*) is a time periodic function. The parameter *A* modulates the amplitude of the periodic input *p*(*t*) and is chosen so that it describes the strength of the input by providing the temporal average of the stimulus (see Methods). We are going to consider a periodic function *p*(*t*) of von Mises type (see Eq (15) in Methods) to explore the effects of varying the input coherence, that is, how concentrated is the input volley around some phases of the cycle. Notice the time constant *τ*_*e*,*i*_ multiplying the periodic input, so that *A* describes the actual effect onto the voltage variable (see Eq (1)).

For this paper, we consider fixed the following parameters for system (1)-(2):

$$\begin{array}{c} \begin{array}{cc} P= & \{\tau_{e}=\tau_{i}=8,\Delta_{e}=\Delta_{i}=1,\eta_{e}=\eta_{i}=-5,\tau_{s_{e}}=1,\tau_{s_{i}}=5,J_{ee}=J_{ii}=0,\\ & J_{ei}=J_{ie}=13\}, \end{array} \end{array}$$ (6)

and we will vary the other ones.

In Fig 1A we present a schematic representation of the E-I neural network described by system (1)-(2) and their excitatory and inhibitory synaptic connections. In Fig 1B we show numerical simulations of the firing rate activity of the network in response to a time dependent input $I_{e}^{ext}\left(t\right)$ using the mean field reduced model (1)-(2) and the full spiking network model. Notice the perfect agreement between both models. We emphasize here the power of this reduced description, which will allow us to perform the mathematical analysis detailed in the next Sections.

We acknowledge that our model has several parameters and we have chosen a particular set P. Although exploring the PING dynamics in the entire parameter space goes beyond the scope of this work, we have performed some numerical experiments to identify the qualitative role played by the most important parameters. Thus, we have observed that the presence of oscillations is a robust phenomenon and parameters mainly shape the features of the periodic orbit. For instance, changes in the synaptic strength *J*_*ie*_ have an impact on the firing rate activity of the I-cells, while changes in *J*_*ei*_ affect the oscillation frequency. Finally, time constants *τ*_*e*,*i*_ and $\tau_{s_{e},s_{i}}$ modulate the oscillation frequency. In the next Section, we discuss in more detail the emergence of oscillations and, in particular, the role of the strength of the tonic currents to E and I cells.

#### 2.1.1 Characterization of gamma oscillations

Before studying the effects of a periodic perturbation onto the E-I network, we characterize the network intrinsic oscillatory activity, that is, when *A* = 0 in (5) and $I_{e,i}^{ext}={\bar{I}}_{e,i}^{ext}$ are tonic currents.

We first identify the values of the external inputs ${\bar{I}}_{e}^{\text{ext}}$ and ${\bar{I}}_{i}^{\text{ext}}$ for which the model (1)-(2) shows oscillations. With strong enough constant drive to the E-cells, the system (1)-(2) transitions from an asynchronous state of low activity (resting) to an oscillatory regime (see for instance the transition at *t* = 20 in Fig 1B). From the viewpoint of dynamical systems, we say that the system undergoes a bifurcation—a qualitative change in the underlying dynamics when a parameter is varied (in our case when ${\bar{I}}_{e}^{\text{ext}}$ or ${\bar{I}}_{i}^{\text{ext}}$ are varied).

**Fig 2:**
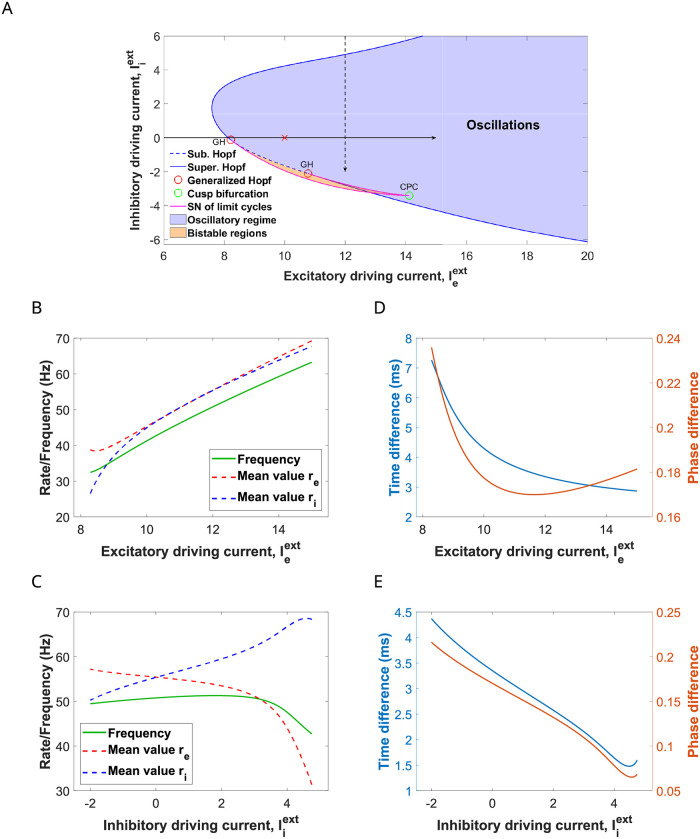
E-I cortical network with oscillatory activity in the gamma range. (A) Two-parameter bifurcation diagram of system (1)-(2) for the excitatory current ${\bar{I}}_{e}^{\text{ext}}$ (*x*-axis) and the inhibitory current ${\bar{I}}_{i}^{\text{ext}}$ (*y*-axis). The solid (resp. dashed) blue curve corresponds to a supercritical (resp. subcritical) Hopf bifurcation curve. Red circles correspond to (codimension 2) Generalized Hopf bifurcations (GH), also known as Bautin bifurcations. Purple curve corresponds to saddle node bifurcation of limit cycles. Green circle corresponds to a Cusp bifurcation of limit cycles (CPC), a point where two branches of saddle-node bifurcations of limit cycles meet tangentially. Oscillations occur in the blue and orange regions. Red cross corresponds to values ${\bar{I}}_{e}^{\text{ext}}=10$ and ${\bar{I}}_{i}^{\text{ext}}=0$ generating the limit cycle considered later on. (B, C) Frequency oscillation (green) and integral mean values of the firing rates *r*_*e*_ (dashed red) and *r*_*i*_ (dashed blue) as a function of (B) tonic excitatory current ${\bar{I}}_{e}^{\text{ext}}$ and (C) tonic inhibitory current ${\bar{I}}_{i}^{\text{ext}}$. See Eq (7). (D, E) Time difference (blue) and relative phase (orange) between inhibition and excitation as a function of (D) tonic excitatory current ${\bar{I}}_{e}^{\text{ext}}$ and (E) tonic inhibitory current ${\bar{I}}_{i}^{\text{ext}}$. In Panels B and D the inhibitory current ${\bar{I}}_{i}^{\text{ext}}$ is set to 0. In Panels C and E the tonic excitatory current ${\bar{I}}_{e}^{\text{ext}}$ is set to 12. Other parameters are as in (6).

We identify two relevant bifurcations in the $\left({\bar{I}}_{e}^{\text{ext}},{\bar{I}}_{i}^{\text{ext}}\right)$-plane to determine the oscillatory states of the system (see Fig 2A). The asynchronous activity states destabilize through Hopf bifurcations (blue curve), which give rise to oscillations (periodic orbits) that can be stable or unstable depending on whether the Hopf bifurcation is supercritical (solid blue curve) or subcritical (dashed blue curve). Along this curve, Generalized Hopf (GH) bifurcations (also known as Bautin bifurcations) occur at the places (red circles) where the Hopf bifurcation changes from supercritical to subcritical (or vice versa) [34]. Moreover, oscillations also disappear (resp. appear) through saddle node (or fold) bifurcations of periodic orbits (purple curve). At these bifurcations, a stable and an unstable periodic orbit collide and annihilate each other (resp. sudden create). Thus, oscillations occur in the blue and orange regions enclosed by the Hopf and saddle node bifurcations. Remarkably, in the orange region, the system presents bistability between asynchronous activity state and oscillatory state. There is also a small region (intersection of orange and blue regions) showing bistability between two different types of oscillations. Indeed, in this region the system presents 3 limit cycles, two of them are stable and one is unstable, in addition to the unstable equilibrium point, corresponding to the asynchronous state. Interestingly, we have found that the size of the bistability region depends on several parameters of the model, in particular, the parameters modeling the mutual interpopulations (*J*_*ei*_, *J*_*ie*_) and recurrent coupling (*J*_*ee*_, *J*_*ii*_). Studying the dynamics in the bistable regions is a topic of interest for understanding the role of oscillations in cognitive tasks. See, for instance, [35] for a study of the response of multistable networks of recurrently coupled spiking neurons to external oscillatory drive and its implications for working memory and memory recall tasks. However, the topic is beyond the scope of this paper and is left for future research.

To characterize the oscillations, we compute their frequency and the integral mean values of the firing rate of both populations, that is,

$$\begin{array}{c} R_{e}≔\frac{1}{T^{*}}\int_{0}^{T^{*}}r_{e}\left(s\right)\;ds\;,\;R_{i}≔\frac{1}{T^{*}}\int_{0}^{T^{*}}r_{i}\left(s\right)\;ds\;, \end{array}$$ (7)

where T* is the oscillation period. The values *R*_*e*_ and *R*_*i*_ in (7) provide an average of the firing rate of the individual E and I-cells, respectively.

Moreover, we compute the time difference between the maximum of the I and E firing rates in one cycle (I to E latency) and the relative phase, i.e., the time difference normalized by the period. These factors provide information about the time it takes for the I-cells to activate and halt the activity of the E-cells, and suggest possible gates for communication in the CTC context [25].

We first consider a fixed value of the external inhibitory current (${\bar{I}}_{i}^{\text{ext}}=0$), and we increase the excitatory one ${\bar{I}}_{e}^{\text{ext}}$ from 6 to 15. This interval covers the progression towards the oscillatory region (see solid arrow in Fig 2A). In Fig 2B we show the frequency of the emerging oscillations as a function of the current injected into the E-cells (green curve). Notice that the frequency is bounded away from zero (characteristic of a Hopf bifurcation) and increases from 30 to 70Hz, which fits in the gamma range. The average firing rate of the E and I cells (see Eq (7)) is close to the macroscopic oscillation frequency for all values of the external current ${\bar{I}}_{e}^{\text{ext}}$ that we have explored. Increasing the external input onto the E-cells causes only a slight shortening of the latency period of inhibition over excitation, especially for oscillations above 40Hz (see Fig 2D).

We also vary the constant external drive onto the inhibitory cells ${\bar{I}}_{i}^{\text{ext}}$ from 6 to -2 (notice that it may take negative values), while the input to the E cells is kept fixed at ${\bar{I}}_{e}^{\text{ext}}=12$. The system is initially in the non-oscillatory region and enters the oscillatory region (see dashed arrow in Fig 2A). Increasing the depolarizing current onto the I-cells ${\bar{I}}_{i}^{\text{ext}}$ does not significantly affect the macroscopic oscillation frequency (the frequency remains close to 50 Hz, except for the final drop around ${\bar{I}}_{i}^{ext}\approx 5$, see green curve in Fig 2C), in contrast with the effect of a depolarizing current onto the E-cells. However, increasing ${\bar{I}}_{i}^{\text{ext}}$ causes a strong effect on the inhibition-to-excitation latency (compare phase difference with Fig 2D), the inactive period after inhibition acts on excitation shortens, and thus the relative phase (see Fig 2E).

In summary, the oscillation frequency is governed by a trade off between the decay time of the inhibitory conductance and the external excitatory drive onto the E-cells. Thus, increasing the depolarizing drive onto the E-cells (${\bar{I}}_{e}^{ext}$) shortens the oscillation period, because excitation can overcome the effect of the inhibition sooner. On the other hand, increasing the depolarizing drive onto the I-cells (${\bar{I}}_{i}^{ext}$), activates the I population faster, thus shortening the I-E latency.

**Fig 3:**
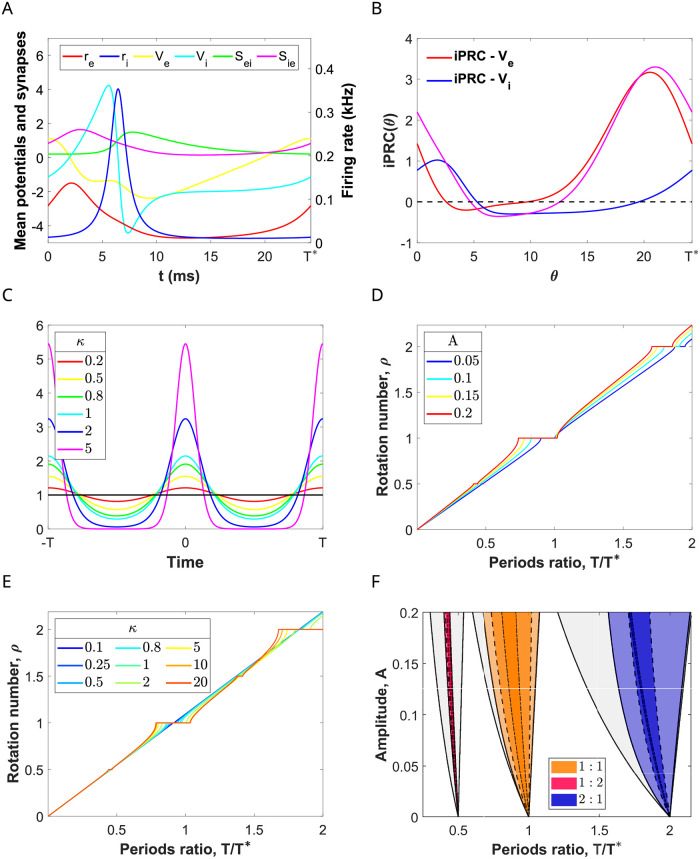
Rotation numbers and phase-locking regions for the forced PING oscillation varying input parameters. (A) Temporal evolution of firing rate, mean membrane potential and synaptic variables over a cycle of a PING oscillation for system (1)-(2) corresponding to the red cross in Fig 2A. (B) Infinitesimal Phase Response Curve (iPRC) for perturbations in the direction of the variables *V*_*e*_ and *V*_*i*_ (red and blue curves, respectively) and the sum of them (purple curve). (C) Von Mises (circular) distribution as a function of the factor *κ* controlling the input coherence. Large values of *κ* result in distributions concentrated around the location *μ* = 0, whereas smaller values lead to broader low-amplitude distributions. The black horizontal line corresponds to the uniform distribution (limit case attained when *κ* = 0). (D, E) Rotation numbers of the stroboscopic map (9) for a von Mises input (15) applied in the direction of *V*_*e*_ and *V*_*i*_, as a function of the ratio between the intrinsic period of the E-I network T* and the input period *T*. (D) Rotation numbers for *κ* = 2 and different amplitude values *A*. (E) Rotation numbers for *A* = 0.1 and different input coherence values *κ*. (F) Arnold tongues computed using the phase reduction corresponding to the 1:1 (orange), 1:2 (purple) and 2:1 (blue) phase-locked states for different input coherence: *κ* = 20, 2, 0.5 corresponding to the regions delimited by solid, dashed, dash-dotted curves, respectively. In grey, we show the corresponding Arnold tongues for pulsatile inputs (*κ* → ∞) obtained analytically. See Methods.

Next, we are going to study the different phase-locking patterns that emerge when the oscillatory neural E-I network receives a periodic external input. To do so, we consider the oscillations that emerge for values ${\bar{I}}_{e}^{\text{ext}}=10$ and ${\bar{I}}_{i}^{\text{ext}}=0$ (red cross in Fig 2A). The oscillation frequency is about 41.26Hz (period T* = 24.234 ms), clearly in the gamma range. Moreover, the time difference between the E and I volley peaks is short (around 4ms, see Fig 2D). Fig 3A shows the evolution of the variables of the system along this periodic orbit. Later in Section 2.6, we will explore another type of oscillations, closer to the Hopf bifurcation curve, showing different features.

### 2.2 Mathematical analysis of phase dynamics

In this Section we describe the mathematical analysis used to study the phase relationship between the neural oscillator described by the low dimensional firing-rate model (1)-(2) and an external time periodic input *g*(*t*) (see Eq (5)).

#### 2.2.1 The phase equation

Consider the case for which the E-I network presents macroscopic oscillatory activity by means of a PING mechanism. Then, for an adequate set of parameters (see previous Section), the system of differential Eqs (1) and (2) has a hyperbolic asymptotically stable limit cycle Γ, of period T*, attracting all nearby orbits. This stable limit cycle is referred to as the oscillator.

Any oscillator can be parametrized by a single phase variable *θ* measuring the elapsed time (modulo T*) from a reference point on the limit cycle, pinpointed as *θ*_0_ (i.e. *θ*(*t*) = *t* + *θ*_0_ mod T*). This point is arbitrary. From now on, we will assume *θ*_0_ = 0 corresponds to the maximum of the mean excitatory voltage *V*_*e*_ on the oscillation. The phase variable *θ* describes uniquely a point on the oscillator via the periodic solution, i.e. *γ*(*θ*(*t*)) = *γ*(*t*) = (*r*_*e*_(*t*), *V*_*e*_(*t*), …, *r*_*i*_(*t*), *V*_*i*_(*t*), …), such that *γ*(*t*) = *γ*(*t* + T*).

When we apply an external periodic input *g*(*t*) of period *T* (see Eq (5)) through the dynamics of the variables *V*_*e*_ and *V*_*i*_, the evolution of the phase variable *θ* is described by the equation,

$$\begin{array}{c} \frac{d\theta }{dt}=1+g\left(t\right)\left(Z_{V_{e}}\left(\theta \right)+Z_{V_{i}}\left(\theta \right)\right), \end{array}$$ (8)

where $Z_{V_{e}}\left(\theta \right)$ and $Z_{V_{i}}\left(\theta \right)$ are the coordinates of the infinitesimal Phase Response Curve (iPRC) *Z* in the direction of the variables *V*_*e*_ and *V*_*i*_, respectively. The function $Z_{V_{e}}$ (resp. $Z_{V_{i}}$) measures the oscillator’s phase shift due to an infinitesimal perturbation applied to the voltage *V*_*e*_ (resp. *V*_*i*_) at different phases of the cycle [31]. Thus, the iPRC $Z_{V_{e}}+Z_{V_{i}}$ measures the oscillator’s phase shift when the perturbation applies to both E and I populations. For, *g*(*t*) = *Ap*(*t*), the positive parameter *A* controls the strength of the input, and it is assumed to be sufficiently small (*A* ≪ 1). See Methods. In Fig 3B, we show $Z_{V_{e}}$ (red curve) and $Z_{V_{i}}$ (blue curve) for the oscillator in Fig 3A (corresponding to the parameter set P in (6) with ${\bar{I}}_{e}^{ext}=10,{\bar{I}}_{i}^{ext}=0$). Notice that the excitatory inputs to the I-cells produce a weaker effect onto the phase compared with inputs to the E-cells (compare the iPRC $Z_{V_{e}}\left(\theta \right)$ and the iPRC $Z_{V_{i}}\left(\theta \right)$ in Fig 3B). Indeed, when the same excitatory input is applied to both populations, the iPRC $Z_{V_{e}}+Z_{V_{i}}$ (pink curve) has a similar shape than the iPRC $Z_{V_{e}}\left(\theta \right)$ (red curve). Thus, the oscillator is almost input insensitive for phases of the cycle corresponding to the activation of the I-volley, (iPRC close to zero) and positive for the rest, achieving its maximum value right before the activation of the E-volley. Later in Section 2.6, we will explore another type of oscillations, closer to the Hopf bifurcation curve, for which the iPRC takes both positive and negative values.

#### 2.2.2 Stroboscopic map and phase-locking

Since the input *g*(*t*) is periodic of period *T*, we use the so-called *stroboscopic map* to study the phase Eq (8). Let Φ_*t*_(*θ*) be the solution of the Eq (8) starting at phase *θ*_0_ at *t* = 0. The stroboscopic map *P* (also known as Poincaré phase map) is defined on the circle and provides the phase of the oscillator after a time *T* (the period of the perturbation), that is

$$\begin{array}{c} \theta_{n+1}=P\left(\theta_{n}\right)≔\Phi_{T}\left(\theta_{n}\right)\;\text{mod}\;T^{*}\;. \end{array}$$ (9)

In general, we cannot find an analytical expression for the stroboscopic map and the computation must rely on the numerical integration of the system. However, in the case of pulsatile inputs, we can provide an analytical expression for it (see Methods).

The dynamics of the stroboscopic map determines the phase-locking between the oscillator and the periodic perturbation. In our context, we say that they are *p*: *q* *phase-locked* if every time the forced oscillator completes *p* revolutions, the oscillatory input completes *q* revolutions. The concept of synchronization is often associated to the 1:1 phase-locking, that is, the external stimulus entrains the oscillator and both oscillate at the frequency of the forcing.

The 1:1 phase-locked states correspond to fixed points of the stroboscopic map *P* in (9), because, after integrating the phase equation for a time *T* (the period of the forcing), the phase of the forced oscillator coincides with the initial one (modulo T*), i.e. *θ* + T* = *P*(*θ*). The relation *p* : 1, consisting in *p* revolutions of the oscillator per one of the stimulus, also corresponds to a fixed point of the stroboscopic map (we must find a solution of *θ* + pT* = *P*(*θ*)). Finally, in the most general framework, *p* : *q* phase-locked states correspond to *q*-periodic points of the stroboscopic map, i.e. solutions of the equation *θ* + pT* = *P*^*q*^(*θ*).

#### 2.2.3 Rotation number and Arnold tongues

To identify the fixed and periodic points of the stroboscopic map *P* in (9) (and therefore, the phase-locked states) we compute the rotation number. The rotation number (denoted by *ρ*) measures the angle on the circle that the map *P* turns on average at each iterate. See Methods for the formal definition. Results in dynamical systems theory [36] guarantee that whenever *ρ* is rational (i.e. *ρ* = *p*/*q* for p,q∈N), there exists a *q*-periodic point of the stroboscopic map (9), that is, a solution of $P^{q}\left(\theta \right)=\theta \;\text{mod}\;T^{*}$, corresponding to a *p* : *q* phase-locking relationship. If, by contrast, *ρ* is irrational, then the orbits of (9) fill densely the circle, and phase-locking does not occur.

We have computed the rotation number for the stroboscopic map (9) of the phase Eq (8) corresponding to the oscillator in Fig 3A (whose iPRC $Z_{V_{e}}+Z_{V_{e}}$ is shown in Fig 3B) and a depolarizing input *g*(*t*) = *Ap*(*t*) of von Mises type (see Eq (15) and Fig 3C), for which we will vary the frequency 1/*T*, the amplitude, controlled by the parameter *A*, and the coherence *κ*.

When *A* = 0, the only *p* : *q* phase-locked states occur at rational values of the frequency relationship, *T*/T* = *p*/*q*. However, as *A* increases, there appear plateaus in the graph of the rotation number as a function of *T*/T*, which is known as *Devil’s staircase*. The plateaus, corresponding to rational values of the rotation number, appear because there is a range of periods *T* close to $\frac{p}{q}T^{*}$ that also give rise to *p* : *q* phase-lockings (see Fig 3D). The easiest detectable plateaus correspond to *ρ* = 1, 1/2, 2 for the range of input frequencies considered. Increasing the amplitude, these plateaus are lengthened and shifted towards higher input frequencies.

In the relative frequency vs amplitude parameter space (*T*/T*, *A*)-plane, a *p* : *q* phase-locking region resembles a leaf or a fang with their tip stuck at *A* = 0, known as *Arnold tongue* [36] (see Fig 3F). The plateaus in the graph of the rotation number are actually slices of such Arnold tongues obtained by fixing the amplitude (see Fig 3D). The shape of these Arnold tongues is strongly linked to the sign of the iPRCs. Indeed, when the iPRC is mainly positive (e.g. the iPRC $Z_{V_{e}}+Z_{V_{i}}$ in Fig 3B), the oscillator can essentially only advance its phase and thus it synchronizes almost exclusively to fast external oscillations, that is, periodic forcing with period *T* < T*. The resulting Arnold tongue is bent to the left (see Fig 3F). This fact justifies why the *p*/*q* plateaus in Fig 3D were shifted gradually to the left (see, for instance, at the 1:1 phase-locked state for different amplitudes).

The computation of the phase-locking regions in Fig 3F was carried out under the weak coupling hypothesis (small *A*). For this reason, the prediction of phase-locking for the full model (1)-(2) might be less accurate as the amplitude increases. Remarkably, computations only show a slight disagreement between the prediction obtained from the phase equation and the solution found by exhaustive search of periodic orbits for the perturbed 8-dimensional system (1)-(2) (see S1 Fig). This outcome guarantees the reliability of the predictions based on the phase reduction method at least for amplitudes with *A* ≤ 0.1.

### 2.3 Increasing the coherence of the periodic input enlarges the frequency range that entrains the network

Beyond varying the input strength and frequency, we have also computed the rotation number and Arnold tongues while varying the input coherence. In this paper, the concept of coherence refers to concentration of the periodic input volley around some phases of the cycle (as in [22]). The input coherence is modulated by the parameter *κ* in the input Eq (15), while the total external drive over a cycle is maintained, that is, the area under the input is independent of *κ* (see Fig 3C and Methods). Moreover, we also consider the limit case (*κ* → ∞), the input then becomes a periodic pulse and the pulses are modeled by delta functions. In this case, the Arnold tongues can be computed analytically. See Methods.

We observe that higher coherent inputs (larger *κ*) give rise to devil’s staircases with larger rational plateaus (compare the red curves with the blue ones in Fig 3E). Moreover, when looking at the Arnold tongues of the largest phase-locking regions in the devil’s staircases (1:1, 1:2 and 2:1), we observe that lower coherent inputs (small *κ*) result in narrower Arnold tongues, enclosed in those with larger *κ* (see Fig 3F). Thus, inputs with higher coherence are capable of entraining the target network for a wider range of frequency values. Noticeably, as input coherence increases, the right boundary of the Arnold tongues is set approximately at a fixed frequency relationship *T*/T* ≈ *p*/*q* independently of the amplitude *A* (notice the right boundary of the 1:1 and 1:2 Arnold tongues for *κ* → ∞ is fairly vertical). Thus, increasing the input coherence enlarges the range of frequencies that entrain the network without shifting it.

In the following Section, we explore whether the type of entrainment that emerges inside the 1:1, 1:2 and 2:1 phase-locking regions for coherent inputs (*κ* = 2) is optimal for communication in the CTC context.

### 2.4 Inputs with higher frequency communicate more effectively

In this Section, we explore the implications of phase-locked states for neuronal communication in the CTC context. In the previous Section, we have identified several phase-locked states between an oscillating E-I network (PING interplay) and a periodic input using the phase reduction (see Fig 3). However, phase-locking is not enough to guarantee effective communication; the phase relationship must satisfy certain conditions. The CTC theory proposes that the phase difference between oscillating neuronal groups is adequately set up for communication if windows for sending and receiving are open at the same time, because then the inputs from the pre-synaptic group will produce changes in the firing rate of the post-synaptic population and communication will occur [8, 9].

To identify how information can be encoded in the input, transmitted and represented in the output of the firing rate of the E-cells, we consider periodic inputs with different amplitudes modulated by the parameter *A* (when *A* = 0, the stimulus is not present) and we measure the changes in the firing rate of the post-synpatic group, either on the average firing rate or in the spike synchronization, as suggested in [21]. If inputs with different amplitudes generate different output responses, then the information encoded in the amplitude of the input will be detected at the output and communication will occur.

Moreover, CTC theory proposes that effective communication depends essentially on the timing of the inhibition and the external input [37]. In the unidirectional setting considered herein (see Fig 1A), if the external input volley reaches the receiving E-population before the inhibition activates, it may elicit a response of the E-cells. Thus the target network will detect changes in the input strength by means of its firing rate and render communication effective. On the other hand, if the input volley arrives to the E-population when the inhibition is present, then such an input may have no effect on the activity of the E-cells and information will not be transmitted to the target network.

In this Section, we provide four measures that will allow us to characterize quantitatively whether the input strength *A* is transmitted to the receiving population, how it is represented by the activity of the E-cells of the target network and how this transmission is related to the phase-locking properties of the entrainment. In particular, we compute the factor *Δτ* (Eq (23)), which measures the time difference between the peaks of the input and the I-volley, and the factors $\Delta \bar{\alpha }$, Δ*α* and Δ*σ* which measure the firing rate response of the E-cells of target network to the input (see Methods). More precisely, $\Delta \bar{\alpha }$ (Eq (24)) provides information about the rate change in the overall firing rate of the E-population over the whole cycle, Δ*α* (Eq (25)) about the rate change in the maximum of the E-volley and Δ*σ* (Eq (26)) about the rate change in the half-width of the E-volley. In our previous work [24], we considered only the factor Δ*α*, but the quantities $\Delta \bar{\alpha }$ and Δ*σ* allow us to complement the information about the changes in the firing rate. Thus, the factor Δ*τ* describes the properties of the phase-locking that emerges, in particular, how close in time is the input from the activation of the I-cells, while the other factors describe how the network firing rate is affected by the changes in the input strength.

**Fig 4:**
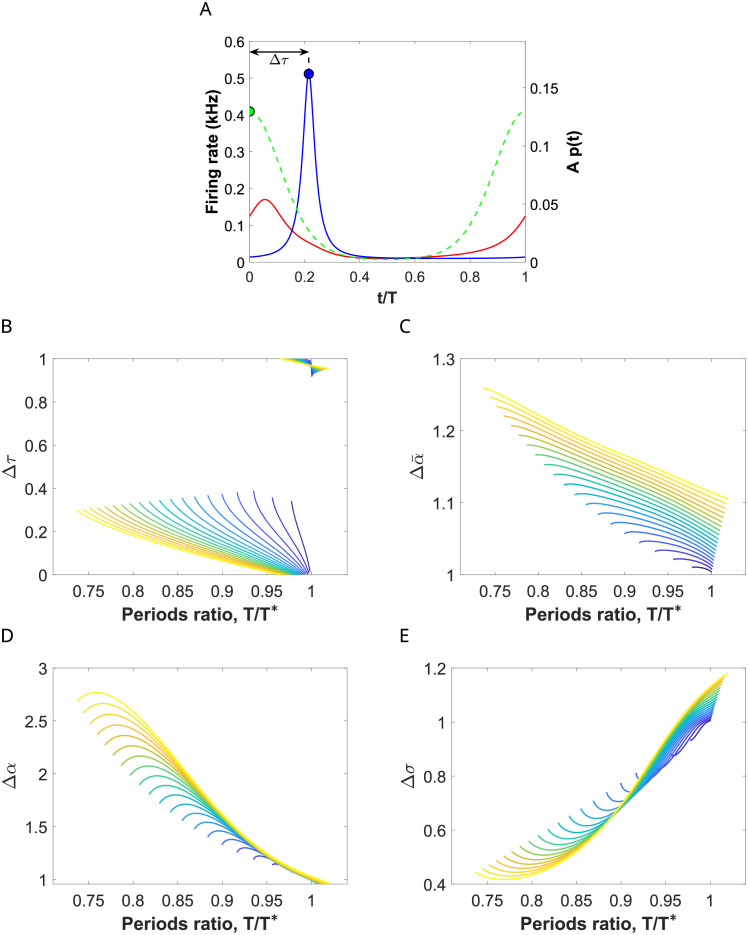
Input effects on the E-cell evoked response for a network entrained by coherent inputs. (A) Evolution of the firing rate variables *r*_*e*_ (red) and *r*_*i*_ (blue) of the perturbed system (1)-(2) with a von Mises input with *κ* = 2 (dashed green) for a representative periodic orbit within the 1:1 phase-locking region. (B-E) Factors describing changes in the E-cell response within this phase-locking region for orbits of the perturbed system (1)-(2) calculated along (equidistant) sections *A* = ct of the corresponding Arnold tongue, indicated by the color of the curve (ranging from dark blue, *A* = 0.01, to yellow, *A* = 0.2, with increments of size 0.01). The factors are: (B) Δ*τ*, describing the timing between inhibition and input volleys (normalized by the input period *T*), (C) $\Delta \bar{\alpha }$, describing the rate change in the averaged firing rate of the E cells, (D) Δ*α*, describing the rate change in the maximum of the firing rate of the E cells, and (E) Δ*σ*, describing the rate change in E-volley half-width. See Methods.

In Fig 4 we show the factors Δ*τ*, $\Delta \bar{\alpha }$, Δ*α*, and Δ*σ* for periodic solutions of system (1)-(2) perturbed with a von Mises input *Ap*(*t*) with coherence *κ* = 2 (see Eq (15)), in the 1:1 phase-locking region (identified in Fig 3), where the existence of these orbits is guaranteed. The factors were computed along constant amplitude values that range between *A* = 0.01 and *A* = 0.2 in the corresponding Arnold tongue.

The entrainment presents different features inside the 1:1 phase-locking region depending on the frequency of the external input. The factor Δ*τ* (see Fig 4A) indicates that when the input frequency is close to the natural gamma cycle (*T*/T* ≈ 1), the input volley reaches the E-population when the I-cells are active (Δ*τ* ≈ 0 or even smaller) and, as the input frequency increases (*T*/T* decreases away from 1), the time by which the input volley precedes the I-population volley increases (Δ*τ* increases up to 0.4). See Fig 4B.

The factor $\Delta \bar{\alpha }$ (Fig 4C) indicates that the increase in the mean firing rate of the E-cells due to changes in the input amplitude *A* is stronger for high frequency inputs (i.e. *T*/T* close to the left boundary) than for inputs with frequencies similar to the natural gamma cycle (i.e. *T*/T* close to the right boundary). However, in all cases we observe a weak modulation of the mean firing rate activity by the input strength *A* (values of $\Delta \bar{\alpha }$ below 1.3).

These differences between input frequencies in the modulation of the output response due to changes in the input strength *A* are more noticeable when looking at the factors Δ*α* and Δ*σ*, describing the shape of the E-volley, that is, the spiking synchronization properties within the target network. For higher input frequencies there is a significant sharpening of the E-volley and therefore an increase in the spike synchronization of the post-synaptic group when *A* increases: the half-width decreases (Δ*σ* much smaller than 1) and the maximum increases (Δ*α* much greater than 1) (see Fig 4D and 4E). For frequencies *T*/T* ≈ 1 (close to the right boundary), the maximum and half-width of the E-volley barely changes when *A* increases (Δ*α* ≈ 1 and Δ*σ* ≈ 1). Indeed, it slightly widens (i.e. the E-cells become less coherent) because the input arrives close to the natural activation of the E-cells. We will see later in Section 2.6 that this effect is more pronounced in the case of oscillations close to the Hopf bifurcation.

Therefore, we have found that changes in the input strength *A* are transmitted and better reproduced at the output by the spike synchronization properties of the E-population (described by both the maximum firing rate and half-width of the E-volley) rather than by the average firing rate *r*_*e*_. Moreover, they are better detected for periodic inputs with higher frequency than the natural gamma cycle. Thus, we establish that communication between input and output is quantified by means of the factor Δ*α* (and, also the factor Δ*σ*), which measures the amplification of the oscillations of the target network (the receiver) due to the periodic perturbation. Thus, we conclude that our results suggest that the entrainment of the network by higher input frequencies than the natural gamma cycle may result in a more effective communication of the information encoded in the input strength.

We acknowledge that we have studied an E-I network driven by a periodic input in the absence of noise, thus our setting is deterministic. However, our predictions on effective communication might be tested in the presence of noise using techniques from information theory. We have run preliminary computations that we include in the S1 Appendix, that seem to agree with our deterministic observations. Further explorations are left for future work. See the Discussion.

We have also explored the type of entrainment that emerges in the 1:2 and 2:1 phase-locking regions (see S3 Fig). In the 1:2 phase-locking region, the network receives two input volleys but responds with only one E-I volley every cycle (see S4 Fig). Thus, only one input volley elicits a response in the target network, while the other one is ignored, due to the activation of inhibition. We observe that the E-volley is modulated in a similar way as in the 1:1 phase-locking region (see panels C-E in S3 Fig).

Finally, in the 2:1 phase-locking region, we find two E/I-volleys per one of the input (see S5 Fig). Thus, the input affects mainly only one E/I-volley (solid line in S3 Fig right), while the second E/I-volley reflects only the intrinsic dynamics of the network and it is slightly modulated by the external input. Interestingly, when the input frequency is decreased, the input modulates both E/I-volleys. Then, in these cases, the low frequency input can be seen as a modulator of the E/I-network rhythm (see Discussion).

### 2.5 Inputs in competition and selective communication

Thus far we have studied how a single periodic external input may entrain the network and how is the communication established between the emitting and receiving populations. In this Section we want to explore how CTC theory implements selective communication. In selective communication the target network receives several stimuli from different sources, but responds only to one of them (the relevant stimulus), while ignoring the others [9].

**Fig 5:**
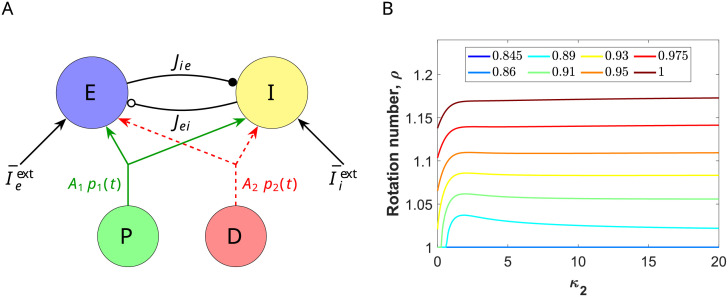
Effect of an identical frequency distractor in the network entrained by the primary stimulus. (A) Schematic representation of an E-I cortical neural network (PING interplay) receiving two oscillatory inputs from different sources: the primary input *A*_1_*p*_1_(*t*) (green circle) and the distractor one *A*_2_*p*_2_(*t*) (red circle). (B) Rotation numbers *ρ* of the stroboscopic map (9) for a perturbation consisting of a primary input and a distractor. Both inputs are modeled by means of a von Mises distribution, have the same amplitude factor *A*_1_ = *A*_2_ = 0.1 and the same period *T* = *T*_1_ = *T*_2_ but phase-shifted. The coherence for the primary is fixed at *κ*_1_ = 2. We vary the distractor coherence *κ*_2_ (*x*-axis) and the period *T*, so that the values *T*/T* (color legend) are distributed along the 1:1 plateau for *κ*_1_ = 2 (the oscillator and the primary stimulus support a 1:1 phase-locking relationship). If *ρ* = 1, the entrainment by the primary stimulus is preserved despite the presence of the distractor, otherwise, it breaks down.

To identify the relevant properties that are involved in selective communication, we probe how the phase-locked states described in the previous Section are affected by the presence of a distractor. In particular, we consider the case where the target network is entrained by a primary periodic input *A*_1_*p*_1_(*t*) of the form (15) with frequency *f*_1_ = 1/*T*_1_ and coherence *κ*_1_ = 2 (coding for the attended stimulus) and we add a second periodic perturbation *A*_2_*p*_2_(*t*) (coding for the distractor) of the form (15) for which we will vary the frequency *f*_2_ = 1/*T*_2_ and the coherence *κ*_2_ (see Fig 5A). We investigate first whether the distractor prevents or not the target oscillatory network to follow the rhythm of the primary stimulus. Second, we investigate how changes in the amplitude of the primary and the distractor affect the output firing rate to determine how the information stored in the amplitude of the primary is transmitted and detected at the output by means of the E-cell evoked response. This approach allows us to identify whether there is a stimulus being detected (attended stimulus) and another ignored and the underlying mechanisms for this selection.

#### 2.5.1 Two input streams of the same frequency

We first consider the case when the distractor has the same frequency as the primary input (i.e, *f*_2_ = *f*_1_ = 1/*T*_2_ = 1/*T*_1_ = 1/*T*), but phase-shifted by *T*/2 so that the two inputs are in anti-phase. We assume that the coherence of the primary is fixed at *κ*_1_ = 2 and we vary the coherence of the distractor *κ*_2_ and the period of both inputs *T*. The input strength is taken *A*_1_ = *A*_2_ = *A* = 0.1 and we consider only those values of *T* for which the primary input entrains the network, that is, the pair (*T*/T*, *A*) lies inside the 1:1 Arnold tongue (see Fig 3F).

Since both inputs have the same frequency, the perturbed system is still *T*-periodic and therefore we can compute the rotation number *ρ* for the stroboscopic map (9) of the phase Eq (8) with *g*(*t*) = *A*_1_*p*_1_(*t*) + *A*_2_*p*_2_(*t*). Recall that if *ρ* ≈ 1, then 1:1 phase-locking is preserved, whereas if *ρ* is away from 1, then the distracting stimulus manages to disrupt phase-locking.

We find that the ability of a distractor to disrupt the entrainment by the primary stimulus depends mainly on the frequency relationship *T*/T* (see Fig 5B). Indeed, for lower values of *T*/T* (on the left-hand side of the 1:1 Arnold tongue), the entrainment is more robust to perturbations by a distractor input (the rotation number remains constant *ρ* = 1). Remarkably, this effect is fairly independent of the coherence of the distractor *κ*_2_. As the input frequency decreases (*T*/T* increases), the entrainment by the primary is significantly affected by the distractor (*ρ* ≠ 1).

For the cases when the entrainment is not disrupted, we will see in Section 2.5.5 that there is a symmetry in the system and the network may switch between the attended stimuli.

#### 2.5.2 Two input streams of different frequency

**Fig 6:**
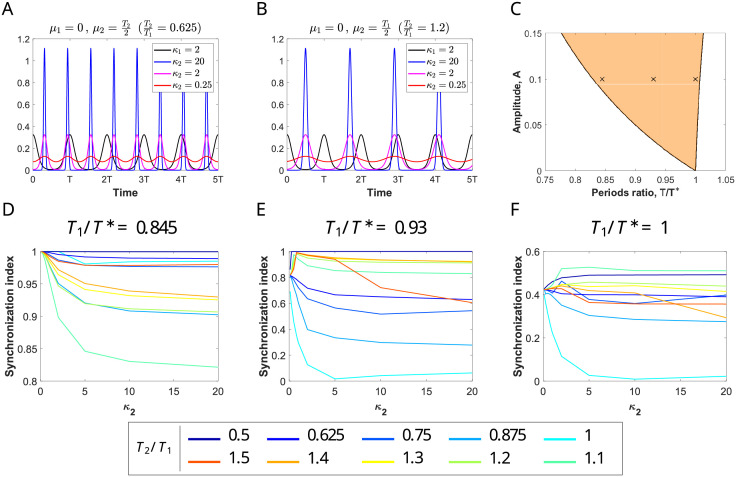
Effect of a non-identical distractor in the network entrained by the primary stimulus. (A, B) Two von Mises inputs of different frequency phase-shifted according to the rule described in Methods. The black curve corresponds to the primary stimulus located about *μ*_1_ = 0 with *κ*_1_ = 2 and period *T*_1_ = *T*. The other curves correspond to a distractor with different coherence values *κ*_2_ indicated in the legend. The distractor is phase-shifted (A) *T*_2_/2 if *T*_2_/*T*_1_ < 1 or (B) *T*_1_/2, otherwise. (C) Arnold tongue corresponding to 1:1 phase-locking between a single von Mises input with coherence *κ*_1_ = 2 (primary input) and the target network. We have selected 3 orbits along the section *A*_1_ = *A* = 0.1 (black crosses) corresponding to *T*_1_/T* = 0.845, 0.93 and 1 (left to right), for which we apply a distractor input of the same strength *A*_2_ = *A* = 0.1. (D, E, F) Synchronization index *r* for the stroboscopic map at time *T*_1_ as a function of the coherence of the distractor *κ*_2_ (*x*-axis) for different values of the periods ratio between inputs, *T*_2_/*T*_1_ (color legend). The distractor frequency can be higher (cold colors) or lower (warm colors) than the primary frequency, being as much twice as fast (dark blue line) or 3/2 times slower (red line).

We now turn to study the effect of a distractor of different frequency than the primary that entrains the network. We fix the parameters *κ*_1_ and *T*_1_ of the primary input *A*_1_*p*_1_(*t*) so that there is a 1:1 phase-locking relationship between the primary and the oscillatory E-I network. Then, we add a second external input *A*_2_*p*_2_(*t*), for which we vary the coherence *κ*_2_ (between 0.01 and 20) and the period *T*_2_ (within the range $\left[\frac{T_{1}}{2},\frac{3T_{1}}{2}\right]$). The distractor is phase-shifted so that both input volleys are as much apart as possible over a cycle (see Fig 6A and 6B and Methods for a precise definition). The amplitude for both inputs is set to *A*_1_ = *A*_2_ = *A* = 0.1.

Notice that in this case the perturbed system (1)-(2) with *g*(*t*) = *A*_1_*p*_1_(*t*) + *A*_2_*p*_2_(*t*) is no longer *T*_1_-periodic, neither the phase Eq (8). However, we still consider the time-*T*_1_ map of the phase equation, which provides a phase over the cycle at each time *T*_1_ (although the evolution of this map depends also on time). See Methods for more details. We compute the synchronization index (SI) *r*, also known as vector strength, for the phases of this map. The synchronization index is a measure of how clustered are the phases on a cycle [38, 39]. Thus, if *r* ≈ 1, the phases are all clustered around the same value, while if *r* ≈ 0 they are scattered around the circle. See S6 Fig.

The frequency relation *T*_1_/T* has been fixed at three different points inside the 1:1 Arnold tongue for *κ*_1_ = 2 along the line *A*_1_ = *A* = 0.1: on the left (*T*_1_/T* = 0.845), in the middle (*T*_1_/T* = 0.93) and on the right-hand side (*T*_1_/T* = 1). See Fig 6C. In Fig 6D-F we show the SI as a function of the frequency relation between the primary input and the distractor (*f*_1_/*f*_2_ = *T*_2_/*T*_1_) and the coherence of the distractor *κ*_2_. We consider that the entrainment of the target network by the primary input is maintained despite the presence of the distractor when SI satisfies *r* > 0.8.

We observe that the entrainment is not disrupted by the presence of a distractor when the relation *T*_1_/T* lies near the leftmost edge of the 1:1 Arnold tongue (see Fig 6D for *T*_1_/T* = 0.845). The distractor can either oscillate faster or slower (within the range $\left[\frac{T_{1}}{2},\frac{3T_{1}}{2}\right]$) or it might even come in the form of sharper or broader pulses (vary *κ*_2_), but it does not prevent the primary input to entrain the oscillator. Notice that the SI remains above 0.8 for all values of *κ*_2_ and frequency relations *T*_2_/*T*_1_.

When *T*_1_/T* lies in the center of the 1:1 Arnold tongue, faster distractors than the primary input (i.e. *T*_2_/*T*_1_ ≤ 1) cause the phase-locking breakdown since the SI falls below 0.8 (see cold color curves in Fig 6E), independently of the distractor coherence *κ*_2_, as long as it is above 1. However, slower distractors than the primary input (i.e. *T*_2_/*T*_1_ ≥ 1) do not disrupt phase-locking significantly. Indeed, the SI remains above 0.8 (see warm color curves in Fig 6E).

Finally, when *T*_1_/T* lies close to the right border of the 1:1 Arnold tongue, the distractor succeeds in completely disrupting the 1:1 phase-locking, irrespective of *κ*_2_ and the frequency relationship *T*_2_/*T*_1_ (see Fig 6F). Indeed, the SI for all values is below 0.5.

In conclusion, if the distractor is coherent enough, what determines the robustness of the entrainment is the frequency relationship *T*_1_/T*. Indeed, the entrainment of the network by the primary input is much stronger (robust to distractors) when the primary has a higher frequency than the natural gamma cycle. In this case, even the more coherent distractors cannot break the 1:1 phase-locking. As the primary input frequency decreases and approaches the natural gamma cycle frequency, the entrainment becomes weaker because, first, faster distractors can break the synchrony and finally, close to the right boundary, any distractor can do so.

In the next Section we will discuss the underlying mechanisms that explain the dependency of the entrainment robustness on *T*_1_/T*.

#### 2.5.3 The role of inhibition

In the previous Section, we observed that the entrainment is more robust for inputs with higher frequencies than the natural gamma cycle. The explanation is related to the phase relationship that emerges between the primary input and the I-volley of the target network. First, notice that in the absence of the periodic primary stimulus, the E-I volley peaks are shifted apart by approx 0.18 phase units when ${\bar{I}}_{e}^{ext}=10$ (see Fig 2D). We emphasize that this difference is maintained more or less constant when we add the entrainment by a von Mises type of input.

Observe that for values of *T*_1_/T* close to left boundary of the 1:1 Arnold tongue, the factor Δ*τ* is close to 0.4 (see Fig 4B), so inhibition is weak when primary input volleys arrive and strong near the distractor volleys, thus suppressing their effects. In conclusion, inhibition shadows the distracting stimulus and phase-locking with the primary is not disrupted. See S7 Fig left.

On the contrary, if *T*_1_/T* is close to right boundary of the 1:1 Arnold tongue, Δ*τ* is close to 0 (see Fig 4B), thus indicating that the peak of inhibition occurs close to the primary input. Therefore, inhibition is low when the distractor volleys reach the E-cells of the target network, thus allowing the distractor to activate E-cells and disrupt phase-locking. See S7 Fig right.

For intermediate values of *T*_1_/T*, only faster inputs disrupt phase-locking (*T*_2_/*T*_1_ < 1), because every time an input volley arrives under sufficiently low inhibition it elicits (indirectly) an I-volley that shadows the effects of subsequent input volleys arriving close in time. Thus, faster distractor inputs are more frequent and are capable to elicit more I-volleys that shadow the effects of the primary volleys and disrupt phase-locking. See S7 Fig middle.

#### 2.5.4 Effects of the disruptor on the E-cell evoked response

We are not only interested in studying whether entrainment is preserved but also how the communication (i.e., how the input strength of the primary input affects the E-cell firing rate respones) is affected by the presence of a distractor. More precisely, in those cases in which the entrainment by the primary input is maintained, we want to explore whether the presence of a distractor affects the response of the E-cells of the target network, and therefore disrupts the communication with the primary. A question that arises is whether the output of the E-I network reflects summation in some sense of the two inputs or whether one dominates over the other.

Exploring in detail the influence of a distractor in the output firing rate and, in consequence, the communication, involves several parameters, such as the relative frequency *T*_1_/*T*_2_ and relative amplitudes *A*_2_/*A*_1_ between both inputs (primary and distractor), together with the relative frequency with respect to the natural gamma cycle. In this Section, we highlight the qualitative role played by the most important parameters and we leave the systematic study for future research.

**Fig 7:**
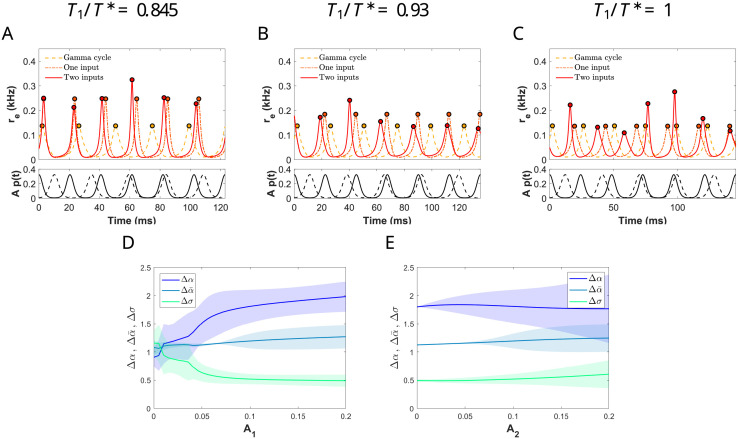
Effects of the disruptor on the E-cell evoked response for some representative trails. Three different types of entrainment inside 1:1 phase-locking region corresponding to (A) *T*_1_/T* = 0.845, (B) *T*_1_/T* = 0.93 and (C) *T*_1_/T* = 1. (Top) Time evolution of the excitatory firing rate *r*_*e*_ for system (1)-(2) in the absence of perturbation (dashed yellow curve), when the network is entrained by the primary input (dashed-dotted orange curve) and when both the primary input and the distractor are present (solid red curve) for a time duration of 6*T*_1_. (Bottom) The primary input (solid black) and the distractor (dashed black) correspond to inputs of von Mises type with *A*_1_ = *A*_2_ = *A* = 0.1, *κ*_1_ = *κ*_2_ = 2 and a frequency relationship *T*_2_/*T*_1_ = 1.2. (D, E) Factors Δ*α*, Δ*σ* and $\Delta \bar{\alpha }$ describing changes in the E-cell evoked response for the case *T*_1_/T* = 0.845 (primary on the left-hand side of the Arnold tongue) and *T*_2_/T* = 1.2*T*_1_/T* = 1.04 (distractor on the right-hand side of the Arnold tongue) when (D) the amplitude of the primary *A*_1_ is varied from 0 to 0.2 while keeping the amplitude of the distractor fixed at *A*_2_ = 0.1 and (E) viceversa. We have computed the mean and standard deviation of these factors over 10 cycles of the primary input.

The main findings are summarized in Fig 7, where we show for some representative examples, the firing rate *r*_*e*_ of the E-cells in the absence of input (yellow dashed curve), when only the primary input is present (orange dash-dotted curve) and when both the primary and the distractor inputs are present (red solid curve). The representative examples chosen are with *T*_1_/T* as in Fig 6 and *T*_2_/*T*_1_ = 1.2, *κ*_1_ = *κ*_2_ = 2.

For values of *T*_1_/T* on the left-hand side of the 1:1 Arnold tongue, the I-volley is in anti-phase with the primary input volley. Thus, the windows for the disruptor to evoke a response are only open during the arrival times of the primary volley. Therefore, depending on the frequency relationship *T*_2_/*T*_1_, we have that, for some cycles, the input volley of the distractor and the primary input almost coincide, thus providing more concentrated input drive to the E-cells and evoking a higher response, while for other cycles, the distractor input volley reaches the E-cells when inhibition is present and the network does not respond to the distractor. See also S7 Fig left and panel A in S8 Fig.

For values of *T*_1_/T* in the middle region of the Arnold tongue, the presence of a distractor decreases on average the evoked response of the E-cells to the primary input, even when the network is still entrained by the primary (compare red solid and orange dash-dotted curves in Fig 7B). The reason is again related to the timing of the I-volley. The time difference between the primary input volley and the I-volley has shortened (see Fig 4B), thus offering more temporal windows (away from the primary input volley) for the disruptor to elicit a response on the E-cells. This causes that for certain cycles the disruptor volley elicits a response ahead of the primary, thus affecting the overall response. See also S7 Fig middle and panel B in S8 Fig.

For the case *T*_1_/T* = 1, the primary input elicits a weak response on the E-cells (compare yellow dashed and orange dash-dotted curves in Fig 7C), while the distractor increases the response in the E-cells (compare red solid and orange dash-dotted curves in Fig 7C). In this case, most of the distractor volleys arrive when inhibition is low, thus causing the activation of the E-cells and affecting the entrainment. See also S7 Fig right and panel C in S8 Fig. In this case, the output firing rate reflects the effects of both primary and distractor inputs.

Next, we take a more systematic approach to assess the contribution of each input to the E-cell evoked response in a particular setting. We consider the case where the primary input has a frequency relationship with the natural gamma cycle that lies on the left-hand side of the Arnold tongue (*T*_1_/T* = 0.845), and we consider a distractor that has a frequency *T*_1_/*T*_2_ = 1.2 (*T*_2_/T* = 1.04, thus, it lies on the right-hand side of the Arnold tongue). Recall that we have seen in Section 2.4 that inputs with higher frequency communicate more effectively, so we will explore whether this is maintained when we add the distractor. We computed the average changes in the maximum firing rate of the E-cells Δ*α*, half-width of the E-volley Δ*σ* and average firing rate $\Delta \bar{\alpha }$ over several cycles in the presence of a distractor with strength *A*_2_ = 0.1 and for varying *A*_1_ (see Fig 7D). We repeated the computations fixing the strength of the primary (*A*_1_ = 0.1) and varying *A*_2_ (see Fig 7E). We can observe that increasing *A*_1_ from 0 (stimulus off) to 0.1 increases significantly the firing rate synchronization within the E-population (described by an increase in Δ*α* and a decrease in Δ*σ*) in the presence of a the distractor (Fig 7D), suggesting that information about stimulus state described by the input strength might be efficiently transmitted. However, increasing the strength of the distractor when the primary input is present affects very little the firing rate of the E-cells both in terms of average firing rate and sharpening of the E-volley (very small variation of the factors Δ*α*, $\Delta \bar{\alpha }$ and Δ*σ* in Fig 7E), suggesting that this input is ignored. Remarkably, in all cases, the average firing rate of the E-cells over a cycle shows little modulation by the strength of the primary and distractor, thus producing little encoding.

This result suggests that the network is capable to detect changes in the strength of a target periodic input, while ignoring simultaneously active distracting periodic inputs, if the frequency of this input is higher than the natural gamma cycle. Again these predictions might be tested in the presence of noise using tools from information theory (see supporting information S1 Appendix for preliminary results and the Discussion).

#### 2.5.5 Selective communication and switching between identical input streams

When the E-I network receives two identical input streams, we have already identified a range of frequencies, for which the target network oscillates coherently (phase-locked) with both of them. Interestingly, the system presents bistability between two possible phase relationships. We want to explore whether these two phase relationships correspond to the network responding differently to each input, suggesting selective communication. Finally, we will study how the network may switch between these two possible phase relationships, thus suggesting a mechanism for switching between attended inputs [16].

**Fig 8:**
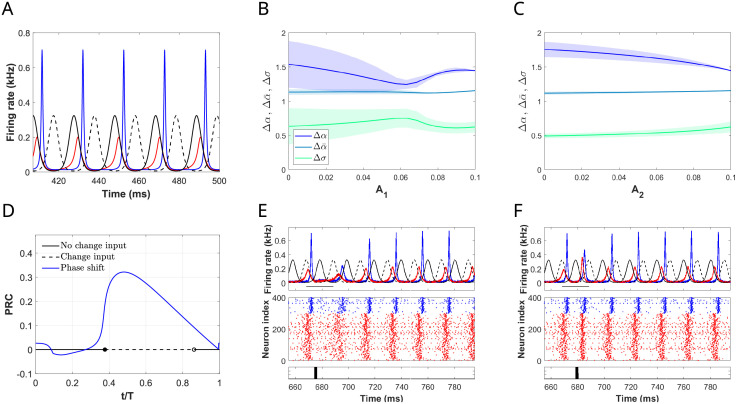
Selective communication and switching between attended stimulus. (A) Time evolution of the excitatory and inhibitory firing rate *r*_*e*_ (red), *r*_*i*_ (blue), respectively for system (1)-(2) receiving two identical inputs of von Mises type in antiphase (*κ*_1,2_ = 2 and *T* = *T*_1,2_ = 0.84T*, *A*_1,2_ = 0.1). We establish that the closest input volley to the E-volley corresponds to the primary input (black solid curve), and the other one to the distractor (black dashed curve). (B, C) Factors Δ*α*, Δ*σ* and $\Delta \bar{\alpha }$ describing changes in the E-cell evoked response (B) when the amplitude of the primary *A*_1_ is varied from 0.1 to 0 while keeping the amplitude of the distractor fixed at *A*_2_ = 0.1 and (C) viceversa. We have computed the mean and standard deviation of these factors over 10 cycles of the primary input. (D) Phase Response Curve (solid blue) obtained by applying square-wave perturbations of amplitude 1.5 and duration 2 ms at different phases of the periodic solution in panel A. The plot also shows if the (entrained) oscillator remains in the same periodic solution before and after the pulse administration (black solid line) or if the oscillator switches to a different solution where the roles of the primary and distractor are exchanged (dashed black line). (E, F) Simulations of the full spiking QIF model showing the response of the network to a square-wave current delivered at two different phases of the cycle: (E) *t*/*T* = 0.3, for which no switching between attended stimuli occurs, and (F) *t*/*T* = 0.5, for which switching occurs. Each panel shows (from top to bottom), for a time interval of 150 ms, the two identical von Mises inputs in antiphase (solid and dashed black) with the mean firing rates of the E-cells (red) and I-cells (blue) of the full spiking QIF model, the corresponding raster plot and the time at which the square-wave pulse is applied. We have integrated the full network of QIF neurons for 1000 ms. At time 200 ms we apply the two inputs of von Mises type. The square-wave pulse is applied at time 200 + 23*T* + *t* ms.

Consider an oscillatory E-I neural network entrained by two identical input streams *A*_*i*_ *p*_*i*_(*t*), *i* ∈ {1, 2} of the form (15) with *A*_*i*_ = 0.1 located in anti-phase (see, for instance, Fig 8A and panel C in S7 Fig left). This situation occurs in the 1:2 Arnold tongue (see Fig 3F and S4 Fig), and similarly, in the case of two identical inputs with a particular period relationship with the natural gamma cycle *T*/T* (those values such that the rotation number *ρ* = 1 in Fig 5B). Notice that in the latter case, the 1:1 phase-locking between the target network and the first input is not disrupted by the addition of a secondary one.

Given a solution corresponding the network being entrained at a particular phase relationship by two identical inputs, we establish that the closest input volley in time to the E-volley corresponds to the primary input and the other one to the secondary (see Fig 8A). We will explore what is the contribution of each input to the output firing rate, by means of modifying the input strength. We vary the strength of the primary input *A*_1_ away from 0.1, while keeping the distractor fixed (*A*_2_ = 0.1). We observe that the synchronization of the E-cells decreases when *A*_1_ decreases from 0.1 (Δ*α* decreases and Δ*σ* increases, see Fig 8B), until the primary input is too weak and the distractor takes over (this corresponds to values of *A*_1_ ≈ 0.06 in Fig 8B). However, when we repeat the same process but keeping the primary input fixed at *A*_1_ = 0.1 and varying the amplitude of the distractor, we observe that the response of the E-cells shows the opposite trend. That is, decreasing the strength of the distractor increases the synchronization properties of the E-cells (Δ*α* increases and the Δ*σ* decreases, see Fig 8C). Based on this observation, we interpret that the network responds to the primary, because the primary amplifies the oscillations of the target network, while the distractor interferes in the communication of the primary (reduces the amplification effect of the primary). For the other phase relationship, the problem is symmetrical and the role of both inputs is reversed. The mean firing rate barely changes in both cases (see $\Delta \bar{\alpha }$ in Fig 8B and 8C).

We study the effects of a brief/short stimulus (a pulse) to the entrained network. Brief stimuli could transiently lengthen or shorten the cycle period (i.e. phase shifting) and force the oscillator to switch phase-locking, thus producing a change in the role played by the inputs. To quantify these effects, we consider the periodic solution for the perturbed 8-dimensional model (1)-(2) with *g*(*t*) = *A*_1_*p*_1_(*t*) + *A*_2_*p*_2_(*t*) and apply a square wave pulse of amplitude 1.5 and duration 2 ms (i.e. brief and short stimulus) to the E-cells. In Fig 8 we show, for a particular case, the change in phase (blue curve) and in the effective input (black solid/dashed line), as a function of the phase *t*/*T* of the entrained oscillator at which the pulse is applied. See Methods. Solid line denotes no change in effective input—the oscillator responds to the same input stream after the pulse—and dashed line denotes a change—the oscillator responds to the other input stream after the pulse. Note that the phase shift is positive almost for all phases, meaning that a pulse typically causes a phase advance (i.e. the E-volley occurs earlier). Notice that there is a small range of phases (between 0.4 and 0.8 approximately) for which a square wave pulse produces a switch in the effective input. This interval corresponds to phases of the cycle for which the I-volley just passed and the next E-volley has not arrived yet (see, for instance, Fig 8F). Notice that the phase shift is close to zero if the pulse arrives at the same time as the I-volley (see, for instance, Fig 8E).

We simulate the full microscopic model to support and illustrate these results. A pulse perturbation of the full microscopic model at phase 0.3 does not entail a change in the effective input (oscillator still responds to the dash-lined input after the pulse is applied in Fig 8E), while at phase 0.5 it does cause a change in the effective input (oscillator responds to the solid-lined input after the pulse is applied in Fig 8F), as predicted by the reduced model.

### 2.6 Oscillations close to the Hopf bifurcation

**Fig 9:**
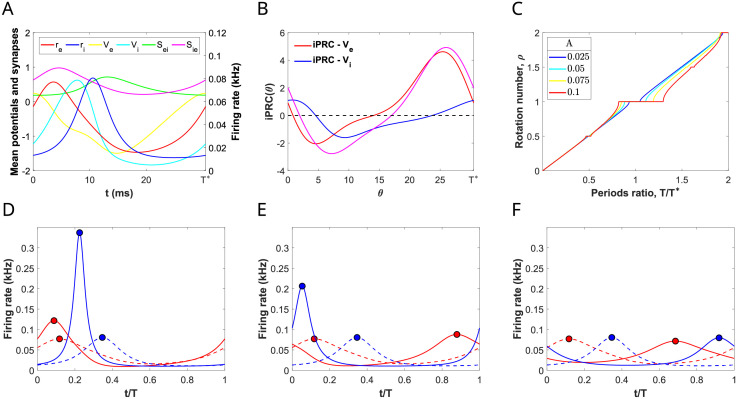
PING oscillations close to a Hopf bifurcation. (A) Temporal evolution of firing rate, mean membrane potential and synaptic variables over a cycle of a PING oscillation for system (1)-(2) corresponding to external current ${\bar{I}}_{e}^{\text{ext}}=8.4$ (close to the Hopf bifurcation curve in Fig 2A). (B) Infinitesimal Phase Response Curve (iPRC) of the cycle in Panel A for perturbations in the direction of the variables *V*_*e*_ and *V*_*i*_ (red and blue curves, respectively) and to both of them (purple curve). Note that the iPRC $Z_{V_{e}}+Z_{V_{i}}$ is both positive and negative. (C) Rotation numbers of the stroboscopic map (9) for a von Mises input (15) with coherence *κ* = 2 applied in the direction of *V*_*e*_ and *V*_*i*_, as a function of the ratio between the intrinsic period of the E-I network T* and the input period *T* and different amplitude values *A*. (D, E, F) Time evolution of the mean firing rates of the E-cells (red) and I-cells (blue) along the unperturbed (dashed curves) periodic orbit for the system (1)-(2) and perturbed (solid curves) with the coherent von Mises input with *A* = 0.05 and relative frequency *T*/T*: (D) 0.9, (E) 1 and (F) 1.07. The period of the oscillators has been normalized to 1.

Thus far we have observed that the timing and shape of the I-volley in the gamma cycle plays a relevant role in implementing several aspects of the CTC theory. To emphasize better this role, we explore how CTC theory implements in a different type of gamma cycle. More precisely, we consider oscillations arising from the PING mechanism but closer to the Hopf bifurcation curve in the parameter space $\left({\bar{I}}_{e}^{\text{ext}},{\bar{I}}_{i}^{\text{ext}}\right)$ (see Fig 2A). Thus, for parameters as in (6) and tonic currents to the E and I-cells ${\bar{I}}_{e}^{ext}=8.4$, ${\bar{I}}_{i}^{ext}=0$, respectively, the system (1)-(2) presents oscillations at a frequency 1/T* = 1/30.5 ≈ 32.78Hz (lower gamma range) of the form shown in Fig 9A. Notice that E/I-volleys are wider and lower for this oscillation than the ones considered along this paper with ${\bar{I}}_{e}^{ext}=10$ (compare with Fig 3A).

Moreover, we also compute the iPRC for this oscillator (see Fig 9B). The iPRC takes both positive and negative values, as opposed to the oscillator with ${\bar{I}}_{e}^{ext}=10$ (compare with Fig 3B). This means that this oscillator may be entrained by either faster or slower periodic inputs. This is the typical behavior for oscillations close to a Hopf bifurcation [31].

We perturbed this oscillator with a periodic input *g*(*t*) = *Ap*(*t*), with *p*(*t*) of von Mises type with *κ* = 2 (see Eq (15)) and applied the phase reduction method described in Section 2.2. The rotation numbers for the stroboscopic map (9) as a function of the relative frequency (*T*/T*) are plotted in Fig 9C for different amplitude values. The visible plateaus, corresponding to rational rotation numbers (*ρ* = *p*/*q*), are centered about the point *T*/T* = *p*/*q* and extend towards both sides. Therefore, the oscillator can be entrained by faster (i.e. *T*/T* < *p*/*q*) and slower inputs (i.e. *T*/T* > *p*/*q*), as expected from the iPRC shape. Notice that the largest plateaus correspond to 1:1 and 2:1 phase-locking (*ρ* = 1 and *ρ* = 2, respectively) and they grow as the amplitude *A* increases.

Since the rotation numbers in Fig 9C have been computed using the phase reduction approach, the phase-locking predictions may not be accurate for the perturbed 8-dimensional mean-field model (1)-(2), specially when the amplitude increases. Indeed, we observe that the actual phase-locked solutions do not extend as far to the right (towards lower input frequencies), rather they extend a little to the left (towards higher input frequencies) than predicted by the phase reduction. Thus, for *A* = 0.05, the phase reduction identifies 1:1 phase-locking for *T*/T* ∈ [0.883, 1.11], but the actual computation finds synchronized solutions for *T*/T* ∈ [0.861, 1.07].

Even if 1:1 phase-locking for low input frequencies does not extend as far to the right as predicted, we still find entrained solutions for inputs with frequencies lower than the network intrinsic frequency (*T*/T* > 1). It is interesting to explore how is the response in the target network for low input frequencies, compared to those with high input frequencies. We look for periodic solutions of the perturbed mean-field model (1)-(2) along the 1:1 phase-locking plateau for *A* = 0.05. For *T*/T* = 0.9 (high input frequency) the entrained oscillator shows a significant increase of the firing rate activity of both E and I-cells (compare solid and dashed lines in Fig 9D). As the frequency of the input becomes more similar to the natural gamma cycle, the E-volley becomes lower and wider (see Fig 9E and 9F for *T*/T* = 1 and *T*/T* = 1.07, respectively). This is because the E-cells activate due to both, the intrinsic properties of the network and the input volley, the latter arriving shortly after the E-cells start to activate but before they can trigger inhibition. Thus, the inputs with low frequencies contribute to widen the E-volley, but without increasing its maximum significantly (the E-cells become less coherent). For larger values of *T*/T* we do not find periodic solutions anymore.

**Fig 10:**
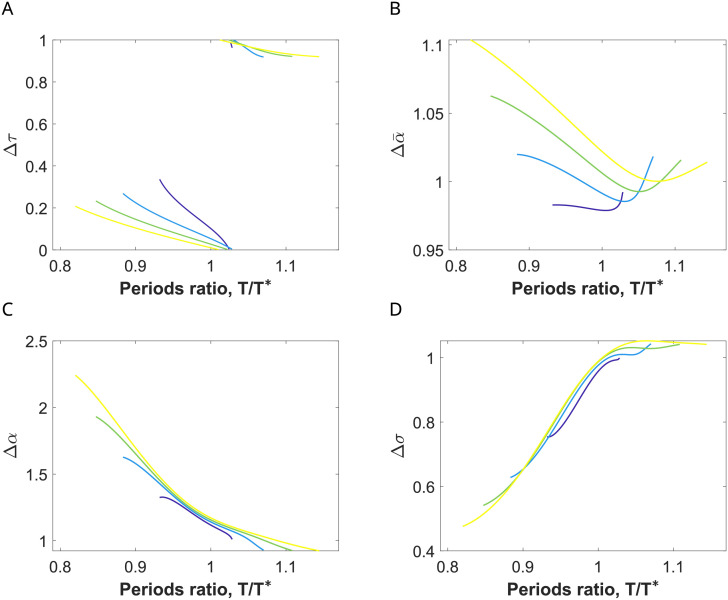
Input effects on the E-cell evoked response for entrained network close to a Hopf bifurcation. Factors (A) Δ*τ*, (B) $\Delta \bar{\alpha }$, (C) Δ*α* and (D) Δ*σ* describing changes in the E-cell response within the 1:1 phase-locking region for orbits calculated along the amplitudes *A* = 0.025 (dark blue curves), *A* = 0.05 (blue curves), *A* = 0.075 (green curves) and *A* = 0.1 (yellow curves). Factors are computed for periodic solutions of the system (1)-(2) perturbed by a von Mises type of input with *κ* = 2. Notice that the periodic solutions are found for smaller intervals than those predicted by the rotation number using the phase reduction (see Fig 9C).

To better identify the aspects related to CTC theory, we compute the factors Δ*τ* and $\Delta \bar{\alpha }$ (as well as Δ*α* and Δ*σ*) introduced in Section 2.4 to measure the timing of the inhibition and the changes in the excitatory firing rate due to the perturbation, respectively. We have computed such magnitudes for the solutions in the 1:1 phase-locking region for amplitudes *A* = 0.025, *A* = 0.05, *A* = 0.075 and *A* = 0.1 in Fig 10). The factor Δ*τ* indicates that the input volley precedes inhibition (0 < Δ*τ* < 0.5) for frequencies higher than the natural gamma cycle and decreases as the input frequency decreases (*T*/T* increases) indicating that the input volley overlaps with inhibition when the input frequency is close to the natural gamma cycle (Δ*τ* ≈ 1 and below 1) (see Fig 10A). Accordingly, the factor $\Delta \bar{\alpha }$ measuring the increase in the overall firing rate also reduces as *T*/T* increases up to 1 and only for the largest amplitudes (see Fig 10B), and the E-volley becomes wider (Δ*α* decreases, see Fig 10C, and Δ*σ* increases, see Fig 10D). Interestingly, for values *T*/T* above 1, the average firing rate increases again (Fig 10B) because the E-volley becomes wider (Δ*σ* is above 1), thus causing a decrease in the firing synchronization within the E-population.

## 3 Discussion

In this paper we have studied several hypotheses of the CTC theory, namely, the conditions for optimal phase-locking so that communication is effective and selective communication. The idea of communication considered in this paper assumes that an oscillatory input from a pre-synaptic group produces changes in the firing rate of an oscillatory post-synaptic group, by means of entraining the target network appropriately. Our approach considers a network of E-I cells oscillating in the gamma range, whose macroscopic dynamics can be exactly described by a low-dimensional mean-field model. In the communication setting of CTC theory, the E-I network receives external time-periodic input from an oscillating pre-synaptic population. We vary the amplitude, frequency and coherence (how concentrated are inputs in a cycle) of the external input and determine the conditions under which phase-locking between the input and the target population occurs. We emphasize that we explore in detail how the phase relationship that emerges contributes to communication, measured as the change in the firing rate of the target network due to the perturbation. We observe that inputs with higher coherence can entrain the network for a wider range of frequencies. As a novelty, we identify the frequency relationship between the input and the natural gamma cycle as a relevant parameter for effective communication. Indeed, inputs with higher frequency than the natural gamma cycle result in a better communication because they produce an amplification of the oscillations of the firing rate of the E-cells of the target network. Thus, information about a stimulus can be encoded in the strength of a periodic input and more effectively transmitted if the input frequency is higher than the natural gamma cycle. Moreover, the information is better transmitted and detected in the synchronization of the post-synaptic population of the E-cells rather than in the average firing rate of the E-population. We then focus on the response of the E-I network when it receives input streams in the gamma frequency range from two different pre-synaptic populations. We find that the entrainment by higher input frequencies is more robust to distractors, even for highly coherent distractors.

We stress that the use of mean field models of low dimension allows for mathematical analysis. In particular, we have identified semi-analytically the oscillatory states in the parameter space by means of the phase reduction formalism, which reduces the study of the dynamics of the full model to a 1-dimensional equation defined on the circle (oscillator). Studying the dynamics on the circle has two clear advantages. On one hand, it drastically reduces the dimensions and, on the other hand, one can take advantage of classical results in dynamical systems theory to describe the dynamics [36]. In particular, we compute the rotation number and the synchronization index to identify the synchronized states and phase-locking patterns. Thus, we can provide equations that describe the boundaries of the phase-locking regions (Arnold tongues) in the parameter space (see Eqs (21) and (22) in Methods), when we vary the frequency, amplitude and coherence of the external input. Even if these equations need to be solved using numerical methods, except for the case *κ* → ∞, these are easier and faster to compute than considering the full model, which would result in more complicated and costly computations (see [24] for the case of Wilson-Cowan equations). Our mathematical analysis confirms that, in general, phase-locking occurs through a tilted Arnold tongue (bent towards higher input frequencies) as observed in computational studies of spiking neurons [18]. Indeed, only faster inputs can entrain the network because the input volley can only evoke an excitatory response and end the gamma cycle if it arrives under sufficiently low inhibition [37].

Besides, we have provided more details about the entrainment by higher frequency inputs. Namely, we have observed that increasing the amplitude of the stimulus enlarges and also shifts the range of frequencies that entrain the network towards lower values, while increasing the coherence enlarges the range without shifting it (see Fig 3D and 3E). Thus, our results confirm semi-analytically previous computational studies on spiking networks claiming that E-cells in the PING target network show preferential phase-locking to periodic inputs with higher coherence. This phenomenon was referred to as ‘coherence filtering’ in [37].

It is hypothesized that E-I networks generating gamma rhythms automatically produce an optimal phase relationship because when a sufficiently strong excitatory pulse is delivered to the network, it generally elicits an I-volley (indirectly in the case of PING). If the frequency of the forcer is approximately the frequency of the receiver, the inhibition from the I-volley wears off just when the next input volley is due to arrive. Thus, the entrainment automatically sets up an optimal phase relationship for CTC [20, 37]. In this paper, we have gone beyond this result and provided a quantitative description of this optimality in terms of the effects in the E-cell evoked response (maximum and coherence) of the target network, as well as the robustness of the entrainment to distractors. In our study we have found that the phase difference is not the same for all frequencies that are capable of entraining the E-I network (in line with [18]), and this has consequences for communication. Indeed, those inputs with higher frequencies precede the I-volley by far, resulting in a strong amplification of the activity of the E-cells in the target network due to input perturbations. Actually, the firing rate of the target network increases mildly compared to the increase in the network coherence (spike synchronization within network). In contrast, those inputs with frequencies similar to the receiver step on the I-volley, thus having a weaker effect onto the activity of the E-cells or, even, decreasing the coherence of the E-cells of the target network. Interestingly, this finding is independent of the coherence of the input. Thus, synchrony of the source network does not necessarily produce higher firing in the target network.

In our study, we have also explored the effects of input frequency and coherence in selective communication, the phenomenon in which a target network entrains and responds to only one of several input streams. This mechanism is conjectured to implement selective attention, the process in which subjects focus on a particular object of the environment while ignoring the others. In this context, experimental studies in visual cortex have shown that when two stimuli are presented in the receptive field of a neuron, it can respond to either input depending on which is attended to [14, 40]. Indeed, selective attention is associated with an increase in coherence of spiking and in spectral power of oscillations in the gamma frequency band [14, 41, 42].

Previous modeling studies [22, 37] have observed that higher coherence and frequency lend a competitive advantage to an oscillatory pre-synaptic neuronal population to entrain a target oscillating network. Here, we have gone a step further since we have explored how these two properties interact with each other and, interestingly, we have observed that coherence affects little, compared to frequency. Indeed, the higher the frequency of the primary stimulus that entrains the network, the more robust to desynchronization by disruptors. The distractor (regardless its coherence) can easily break the synchronization of the network with a low frequency primary input, but not for a high frequency one. When the primary input has a much higher frequency than the natural gamma cycle (leftmost regions of the 1:1 Arnold tongue), the position of the I-volley in the cycle makes the entrainment more robust since most of the distractor volleys arrive when inhibitory cells are active. In contrast, for input frequencies similar to the natural gamma cycle (rightmost regions of the 1:1 Arnold tongue), the position of the I-volley lies close to the primary volleys, thus some of the distractor volleys arrive when inhibition is low, eliciting a response of the E-cells that disrupts synchronization with the primary, even for low coherence distractors. This result is in agreement with the experimental results in the visual cortex [40], where V1 sites processing relevant stimuli have its gamma peak frequency higher than the irrelevant V1 sites and higher than the target sites in V4.

As a novelty, we are able to identify not only the effects of the disruptor regarding the phase-locking but also on the evoked response of the E-cells (see Section 2.5.4). We have shown examples where the information encoded in the strength of the input with high frequency is still transmitted to the output firing rate of the E-cells, because the disruptor does not affect the firing rate properties of the E-cells, thus preserving effective communication.

Our results are obtained for a deterministic system, but might provide insight into causal interpretation of results in systems with noise based on tools from information theory and correlations [43, 44]. We present some preliminary computations of mutual information in the supporting information, but more elaborated techniques such as partial information decomposition could be used to elucidate how much information about a secondary stimulus (encoded in the strength of the distractor input) is present in the firing rate of the E-cells and how is the interaction with the primary input, thus exploring the sinergy and redundancy in the information. This could be an interesting subject of research for future work, as well the study of other settings with more than two inputs to identify and quantify more complex interactions and explore other communication strategies such as multiplexing [45–47].

We have also explored how phase shifting can act as a mechanism for stimulus selection. In line with [25], an appropriate optogenetic pulse applied at the right phase can cause switching between the attended inputs. Our results show that the appropriate phases are after the inactivation of the I-volley but before the activation of the E-volley.

The mathematical formalism based on the phase equation used herein requires that the inputs received by the target network are weak, particularly, because a strong perturbation may displace and keep the trajectory far away from the limit cycle [31, 33]. In our study, during the time between input volleys, the displaced trajectories relax back to the original unperturbed oscillator (see panel B in S2, S4 and S5 Figs), therefore allowing for a good prediction based on the phase reduction. Indeed, the predictions on synchronized states obtained from the phase reduction in Figs 3 and 9 were validated computing the corresponding periodic orbit for the reduced mean-field model (1)-(2), showing very good agreement. In addition, we emphasize that in our previous study on Wilson-Cowan equations [24] we did not assume that inputs were weak, and we detected regions in the parameter space showing bistability between synchronous and asynchronous regimes, as well as between different types of entrainment. The method used herein was not designed though to detect bistability in the forced network, therefore the existence of bistability or other dynamical features in the strong coupling regime remains a challenging topic for further studies. Techniques based on the phase-amplitude description might be useful to tackle larger amplitude perturbations [48–50].

Changes in excitability are generated by the interaction between excitation and inhibition, and the frequency and phase relationship between E and I volleys can be tuned by means of changes in tonic drive. In our previous work with Wilson-Cowan equations [24], the dynamics of the oscillating firing rates of the E and I populations resembled those of the E-I network considered herein when ${\bar{I}}_{e}^{ext}=8.4$. Namely, the E and I volleys are less coherent and they spread along the whole cycle (see Fig 9A). Moreover, the oscillation frequency of the network falls in the lower range of gamma oscillations (around 32Hz). Notice that, in this case, the oscillations are close (in parameter space) to a Hopf bifurcation (see Fig 2A) and the iPRC is of Type II (taking both positive and negative values). Thus, as opposed to the case with ${\bar{I}}_{e}^{ext}=10$, the target oscillating network can synchronize to both faster and slower incoming periodic inputs (see Fig 9B). Noticeably, in this case the prediction by the phase reduction is less accurate for the slowest inputs. This case makes clear the different effect of lower and higher frequency inputs than the natural gamma cycle in the activity of the target network. Indeed, higher input frequencies tend to increase both the firing rate and coherence of the network while lower ones tend to make it less coherent (see Fig 10).

We acknowledge the fact that gamma-band rhythms observed in experimental data do not show regular oscillatory behavior, but they are rather irregular and episodic [51, 52]. In [21] the authors consider an E-I network showing irregular and episodic gamma rhythms and observe that this assumption enables a target population to be correlated to two independent sources that differ either in frequency or in phase. Notice that in this case correlations are used to measure entrainment and synchronization between neural populations. We believe that the conclusions obtained for regular oscillations can though provide the substrate to explain several mechanistic phenomena in the irregular case. For instance, we have observed that it is possible to switch the entrainment between two different sources by means of pulses delivered at the proper phases of the cycle. This could be an interesting subject of research for future work.

In this paper we have mainly focused on the interactions between two neuronal groups oscillating in the gamma range, but we have not discussed the interaction of gamma with other rhythms, namely beta (13–30 Hz), alpha (8–13 Hz) and theta (4–8 Hz) [35, 37]. The interplay between different frequency rhythms may definitely have an important role for communication and cognition [9, 37, 53–55] and the methods discussed herein may contribute to unveil possible mechanisms. Namely, a thorough study of the solutions emerging in the p:1 phase-locking regions, where the input is *p* times slower than the intrinsic oscillatory frequency. We have studied the 2:1 phase-locking regions and observed that the slow input modulates the E/I-volleys of the gamma cycle, in a similar or different way depending on the frequency relationship between both rhythms *T*/T* and the input coherence (see S3 Fig right and S5 Fig).

Finally, we would like to discuss other aspects of CTC that have not included in this study. In the E-I network considered, even if the excitatory inputs synapse to both E and I cells, the effect of the input on the activity of the I-cells is mild compared to the E-cells. However, exploring the input effects of inputs onto the inhibitory cells (see for instance [56]) in a different PING regime or in the case of gamma oscillations generated by means of the ING (Interneuron Network Gamma) mechanism [4], will provide insight into the dynamics of oscillations and transmission of information. For example, input to the I-cells have been proposed as a mechanism for phase-shifting in models of cortical networks [25]. Finally, our setting can be extended to networks of two populations to explore bidirectional communication [43, 10, 29, 57, 58] and delays [44] and its main differences with the unidirectional communication setting explored in this paper.

## 4 Methods

### 4.1 Neuron models

We consider a population of *N* neurons with all-to-all coupling, subdivided into a population of *N*_*e*_ excitatory and *N*_*i*_ inhibitory cells, whose individual voltage dynamics are modeled by a Quadratic Integrate-and-Fire (QIF) model [59], i.e.,

$$\begin{array}{c} \tau {\dot{V}}_{j}=V_{j}^{2}+\eta_{j}+I_{j}\;,\;\text{if}\;V_{j}\geq V_{\text{th}}\;\text{then}\;V_{j}=V_{\text{reset}}\;,\;j=1,\ldots ,N\;, \end{array}$$ (10)

where *V*_*j*_ is the voltage of the *j*-th neuron, *τ* is the time constant, *η*_*j*_ is a constant bias current and *I*_*j*_ is an input current accounting for external (time-dependent) stimuli and the excitatory/inhibitory synapses of the network. The threshold and reset voltages are taken *V*_th_ = −*V*_reset_ → ∞, (in numerical simulations, we will set them to *V*_th_ = −*V*_reset_ = 500). Heterogeneity in the system is introduced by assuming that the parameters *η*_*j*_ are distributed according to a Lorentzian distribution with half-width Δ and centered at $\bar{\eta }$,

$$\begin{array}{c} L\left(\eta \right)=\frac{1}{\pi }\frac{\Delta }{{\left(\eta -\bar{\eta }\right)}^{2}+\Delta^{2}}\;. \end{array}$$ (11)

The input current *I*_*j*_ consists of a common external input for all neurons and synaptic current due to recurrent connexions in the circuit. For the excitatory ensemble, the input is expressed as

$$\begin{array}{c} I_{e}=I_{e}^{\text{ext}}+\tau_{e}S_{ee}-\tau_{e}S_{ei}\;, \end{array}$$ (12)

while for the inhibitory one as

$$\begin{array}{c} I_{i}=I_{i}^{\text{ext}}+\tau_{i}S_{ie}-\tau_{i}S_{ii}\;. \end{array}$$ (13)

We have omitted the subindex *j* (labeling each neuron in the population) because all the neurons in the same population receive the same input current. The subscript indicates whether it corresponds to the excitatory (E) or the inhibitory (I) population. The variable *S*_*ab*_ models the synaptic current from the pre-synaptic population *b* to the post-synaptic population *a*, where *a*, *b* ∈ {*e*, *i*}. The dynamics of the synaptic currents are described by a linear differential equation of the form

$$\begin{array}{c} \tau_{s_{b}}{\dot{S}}_{ab}=-S_{ab}+J_{ab}r_{b}\;, \end{array}$$ (14)

where *a*, *b* ∈ {*e*, *i*}. Here, $\tau_{s_{b}}$ is the synaptic time constant of population b, *J*_*ab*_ the synaptic strength from population *b* to population *a*, and *r*_*b*_ the firing rate of population *b*, which is computed as

$$\begin{array}{c} r_{b}\left(t\right)=\frac{1}{N_{b}}\sum_{n=1}^{N_{b}}\sum_{k}\delta \left(t-t_{k}^{n}\right), \end{array}$$

where *δ*(*t*) is the Dirac delta function and $t_{k}^{n}$ are the firing times of neuron *n*.

In [26, 29], one can find the details of the derivation of the reduced mean field model (1)-(2) that provides an exact description (in the thermodynamic limit) of the macroscopic quantities of the spiking model described above, namely, the mean firing rate *r*_*a*_ and membrane potential *V*_*a*_.

### 4.2 Inputs

To model the excitatory input drive to the E and I cells, $I_{e}^{ext}\left(t\right)$ and $I_{i}^{ext}\left(t\right)$, respectively (see Eq (5)), we consider a periodic function *g*(*t*) = *Ap*(*t*), for which we can modulate its coherence, that is, how synchronized are the spikes of the pre-synaptic population providing periodic input. To do so, we use the von Mises probability density function (also known as the circular normal distribution) and define the *T*-periodic function *p*(*t*) as

$$\begin{array}{c} p\left(t\right)=\alpha \frac{e^{\kappa \;\text{cos}(\frac{2\pi }{T}\left(t-\mu \right))}}{TI_{0}\left(\kappa \right)},\;\text{for}\;t\in \left[0,T\right), \end{array}$$ (15)

where *I*_0_(*x*) is the modified Bessel function of order 0. The parameter *μ* is the mean and *κ* is the temporal coherence (*κ* = 0 corresponds to a uniform distribution and as *κ* increases the distribution becomes more concentrated about the angle *μ*. In the limit case, *κ* → ∞, the distribution becomes a delta function). See Fig 3C. The parameter *α* is chosen as *α* = *T* so that the temporal average over one period is 1. That is,

$$\begin{array}{c} \frac{1}{T}\int_{0}^{T}p\left(t\right)dt=1. \end{array}$$ (16)

When only one input is present, we fix *μ* = 0 and we vary the coherence *κ* and the period *T*.

We also consider the case when the E and I populations receive two input streams of the form (15), one referred to as the primary stimulus and denoted by *A*_1_*p*_1_(*t*), and the other as the distractor and denoted by *A*_2_*p*_2_(*t*). The mean, temporal coherence and period of the *i*-th input will be denoted by *μ*_*i*_, *κ*_*i*_ and *T*_*i*_, respectively.

When both inputs have the same period (*T*_1_ = *T*_2_ = *T*), we place the second input to be in antiphase with respect to the first one, that is *μ*_2_ = *T*/2. However, when the periods are different, the last criterion is not well-defined. Therefore, we implement the following rule: we place the peak of the input with largest period between two consecutive peaks of the input with shortest period. That is, if *T*_2_ ≥ *T*_1_, then $\mu_{2}=\frac{T_{1}}{2}$, whereas if *T*_1_ > *T*_2_, $\mu_{2}=\frac{T_{2}}{2}$.

### 4.3 Phase reduction

For an oscillating E-I network, the 8-dimensional system (1)-(2) has a hyperbolic attracting limit cycle Γ of period T*. The limit cycle can be parametrized by the phase variable *θ*, such that *θ*(*t*) = *t* + *θ*_0_(mod T*) and there exists a function $\gamma :\left[0,T^{*}\right)\to R^{8}$ such that

$$\begin{array}{c} \gamma \left(\theta \left(t\right)\right)=\left(r_{e}\left(t\right),V_{e}\left(t\right),S_{ee}\left(t\right),S_{ei}\left(t\right),r_{i}\left(t\right),V_{i}\left(t\right),S_{ie}\left(t\right),S_{ii}\left(t\right)\right), \end{array}$$

parametrizes the periodic orbit Γ.

We apply an external periodic input *g*(*t*) to the limit cycle Γ in the direction given by the vector $v\in R^{8}$. If the external periodic drive *g*(*t*) is weak, i.e. |*g*| ≪ 1, we can reduce the study of the dynamics of the perturbed system to a single equation for the phase variable, given by

$$\begin{array}{c} \frac{d\theta }{dt}=1+g\left(t\right)\left[Z\left(\theta \right)\cdot v\right], \end{array}$$ (17)

where the function $Z:\left[0,T^{*}\right)\to R^{8}$ is the infinitesimal Phase Response Curve (iPRC). The iPRC measures the oscillator’s phase shift due to an infinitesimal perturbation applied at different phases of the cycle [31]. Notice that *Z*(*θ*) is a vector of 8 components, thus, the *i*-th component (*Z*_*i*_) corresponds to the phase shift due to an infinitesimal perturbation applied in the direction of the *i*-th variable (i.e. $v={\vec{e}}_{i}$). It is well known that the iPRC is the periodic solution of the so-called Adjoint Equation [31], given by

$$\begin{array}{c} \frac{dZ}{dt}=-M^{T}(\gamma (t))\;Z\;, \end{array}$$ (18)

subject to the normalization condition

$$\begin{array}{c} Z\left(t\right)\cdot \frac{d}{dt}\left(\gamma \left(t\right)\right)=1,\; \end{array}$$ (19)

where the matrix *M*(*γ*(*t*)) is the linearization of the system (1)-(2) around the limit cycle Γ.

### 4.4 Rotation number

The existence of periodic points for the stroboscopic map (9) defined on the circle is given by a well known result in the context of circle maps [36, 60]. Let $f:S^{1}\to S^{1}$ be a map on the circle, orientation preserving and regular enough, then the *lift* of *f* is a continuous function $\bar{f}:R\to R$ that satisfies $\pi \left(\bar{f}\left(x\right)\right)=f\left(\pi \left(x\right)\right)$, where *π*(*x*) = *x* mod T* (i.e. the phase on the circle). Then, the *rotation number* *ρ* of the map *f* is defined as

$$\begin{array}{c} \rho \left(f\right)≔\underset{n\to \infty }{\text{lim}}\frac{{\bar{f}}^{n}\left(x\right)-x}{n},\; \end{array}$$ (20)

for any x∈R. Importantly, the limit above always exists and does not depend on the initial point *x*, nor the lift $\bar{f}$ chosen.

There are two important results to characterize the dynamics of the map *f* using the rotation number. If *ρ* is rational (*ρ* = *p*/*q* with p,q∈N), then there exists a *q*-periodic point of the map *f*, that is, a solution of *f^q^*(*θ*) = *θ* mod T*. If, by contrast, *ρ* is irrational, then the orbits of *f* fill densely the circle and there are no periodic points.

We use the numerical procedure described in [61] to compute the rotation number of the stroboscopic map (9) in Figs 3D, 3E, 5B and 9C. We have used *N* = 750 iterations, and the error is of order 10^−6^.

### 4.5 Arnold tongues

The boundaries of the Arnold tongues correspond to the locus of a saddle node bifurcation [62] of the stroboscopic map (9) in the (*T*, *A*)-parameter space.

The saddle node bifurcation curves, corresponding to the boundaries of the *p* : *q* Arnold tongue, can be numerically computed by identifying the points (*θ*, *T*, *A*) such that the following two conditions hold:

$$\begin{array}{c} \left\{ \begin{array}{cc} P^{q}\left(\theta \right) & =\theta +pT^{*},\\ \frac{\partial P^{q}}{\partial \theta }\left(\theta \right) & =1. \end{array}\right. \end{array}$$ (21)

Notice that, although not specified, the stroboscopic map *P* depends on the parameters *T* and *A*. Moreover, since we take modulus T*, we can omit the term pT* in the above equation.

The equations above describe a 1-dimensional curve in $R^{3}$, that can be computed using a continuation method. The continuation method is a numerical procedure by which one can find a curve in $R^{n}$ defined implicitly by a set of (nonlinear) equations *G*(*w*) = 0, where $G:R^{n+1}\to R^{n}$ is regular enough. Notice that this corresponds to a system with *n* nonlinear equations with *n* + 1 variables. The method to find the curve combines a tangent-like approximation as in the Keller’s (pseudo-arclength) method and a modified Newton method (based on a minimization problem with a restriction) to refine it. More precisely, starting from an initial solution *w*_0_ lying on the curve, *G*(*w*_0_) = 0, the method consists in making a prediction for the next point on the curve by moving along the tangent line to the curve at the point *w*_0_. Then, we correct the approximate point successively by means of a modified Newton method. Since the number of variables is higher than the number of equations, we need to impose the additional condition that the norm of the correction must be a minimum. We solve this problem using Lagrange multipliers. The details of the method can be found in [61, 63].

In our case, the problem consists of three variables (*θ* and the parameters *T* and *A*, after allowing them to evolve according to $\dot{T}=\dot{A}=0$) and the two Eq (21). Let *w* = (*θ*, *T*, *A*) be the unknowns and ${\tilde{\Phi }}_{t}\left(\theta ,T,A\right)$ be the extended flow associated to the phase Eq (8) when *T* and *A* are treated as variables, and $\tilde{P}$ the associated stroboscopic map. Therefore, we define the function *G* as

$$\begin{array}{c} G\left(w\right)=G\left(\theta ,T,A\right)=(\begin{array}{c} {\tilde{P}}^{q}\left(\theta ,T,A\right)-\theta \\ \frac{\partial {\tilde{P}}^{q}}{\partial \theta }\left(\theta ,T,A\right)-1 \end{array})=(\begin{array}{c} {\tilde{\Phi }}_{qT}\left(\theta ,T,A\right)\;\;\text{mod}\;T^{*}-\theta \\ \frac{\partial {\tilde{\Phi }}_{qT}}{\partial \theta }\left(\theta ,T,A\right)\;\;\text{mod}\;T^{*}-1 \end{array})\;. \end{array}$$ (22)

To start the method we must provide an initial seed provided by the rotation number. Notice that the Newton method requires to compute the differential of the map *P*, which will be computed using variational equations [63]. Moreover, variational equations involve the computation of the derivative of the iPRC, which is only known numerically for a discrete set of points. Taking advantage of the periodic behaviour of the iPRC, we have used the FFT algorithm to compute the derivatives efficiently.

In the case of pulsatile inputs (*κ* → ∞), the input *p*(*t*) becomes a sum of delta functions, i.e.

$$\begin{array}{c} p\left(t\right)=T\sum_{i\geq 0}\delta \left(t-t_{i}\right), \end{array}$$

where *t*_*i*_ = *t*_0_ + *iT*, 0 ≤ *t*_0_ ≤ *T*. The factor *T* is included because *p*(*t*) was chosen to have average 1 over one period (see Eq (16)), i.e.

$$\begin{array}{c} \frac{1}{T}\int_{0}^{T}p\left(t\right)dt=\frac{1}{T}T\int_{0}^{T}\delta \left(t-t_{0}\right)dt=1. \end{array}$$

Thus, the stroboscopic map at time *T* given in Eq (9) can be written explicitly as:

$$\begin{array}{c} \theta_{n+1}=\theta_{n}+T+A\;T\;Z_{ei}\left(\theta_{n}\right), \end{array}$$

where $Z_{ei}=Z_{V_{e}}+Z_{V_{i}}$. Therefore the map has fixed points (modulus T*) as long as *θ*_*n*+1_ = *θ*_*n*_ + T*, which yields

$$\begin{array}{c} AZ_{ei}\left(\theta_{n}\right)=\frac{T^{*}}{T}-1. \end{array}$$

Denoting *Z*_*max*_ and *Z*_*min*_ the maximum and minimum values of the iPRC *Z*_*ei*_, respectively, we have that the left-hand side and right-hand side borders of the 1:1 Arnold tongue are given, respectively, by the curves

$$\begin{array}{c} \frac{T^{*}}{T}=1+AZ_{max},\;\frac{T^{*}}{T}=1+AZ_{min}. \end{array}$$

Similarly, using that *p* : *q* phase-locking corresponds to *q*-periodic orbits of the stroboscopic map *P* (equivalently, fixed points of the map *P*^*q*^) after *p* turns, we need to impose the condition

$$\begin{array}{c} \theta_{n}+pT^{*}=\theta_{n}+q\left(T+A\;T\;Z_{ei}\left(\theta_{n}\right)\right). \end{array}$$

Thus, the curves corresponding to the left-hand side and right-hand side borders of the *p*: *q* Arnold tongue are given, respectively, by the expressions

$$\begin{array}{c} \frac{T^{*}}{T}=\frac{q}{p}\left(1+AZ_{max}\right),\;\frac{T^{*}}{T}=\frac{q}{p}\left(1+AZ_{min}\right). \end{array}$$

### 4.6 Measures of phase-locking and E-cell evoked response

We present four magnitudes that contribute to the description of the phase relationship between the input and the entrained network and describe the changes in firing rate of the E-cells due to the periodic perturbation. These magnitudes are computed for *qT*-periodic orbits of the perturbed system (1)-(2) with a given function *g*(*t*) = *Ap*(*t*) of period *T*, whose existence is guaranteed in the *p* : *q* phase-locking regions.

The magnitude Δ*τ* computes the normalized time difference between the maximum of the I-volley *r*_*i*_ and the maximum of the external perturbation *p*(*t*), that is,

$$\begin{array}{c} \Delta \tau =\frac{t_{\text{inh}}-t_{p}}{T}\;, \end{array}$$ (23)

where *t*_inh_ and *t*_*p*_ denote the time where the maximum of *r*_*i*_(*t*) and the perturbation *p*(*t*) are achieved over a cycle of period *T*, respectively. Notice that if Δ*τ* lies in the interval [0, 0.5), then inhibition follows the input, which may allow an increase of the activity of the E-cells of the target network. In contrast, if Δ*τ* lies in the interval [0.5, 1), then inhibition precedes the input and the latter may be ignored, bringing on a small or negligible effect onto the activity of the E-cells of the target population.

In the 1:2 phase-locking region, where there are two peaks of the perturbation *p*(*t*) per one of the inhibitory firing rate *r*_*i*_(*t*), Δ*τ* measures the distance between the peak of *r*_*i*_(*t*) and the preceding input peak (see, for instance, panel A in S3 Fig). Notice that Δ*τ* lies in the interval [0, 1). If Δ*τ* > 0 but close to 0, the inhibition immediately follows the first peak and precedes by far the second one. If Δ*τ* < 1 but close to 1, inhibition immediately precedes the second peak and follows by far the first one. For intermediate values of Δ*τ* ≈ 0.5, the I-volley is equidistant from both input peaks.

The effects of the perturbation onto the activity of the E-cells of the target population can be measured as well. We define $\Delta \bar{\alpha }$ as the ratio between the time-average (over a single period [0, *qT*)) of the excitatory activity for the perturbed case (*A* ≠ 0), ${\bar{R}}_{e}^{A}$, and the time-average of the excitatory activity for the unperturbed case (*A* = 0), ${\bar{R}}_{e}^{0}$, in the following way

$$\begin{array}{c} \Delta \bar{\alpha }=\frac{{\bar{R}}_{e}^{A}}{{\bar{R}}_{e}^{0}}\;,\text{with}\;{\bar{R}}_{e}^{A}=\frac{1}{qT}\int_{0}^{qT}r_{e}^{A}\left(s\right)\;ds\;\text{and}\;{\bar{R}}_{e}^{0}=\frac{1}{T^{*}}\int_{0}^{T^{*}}r_{e}^{0}\left(s\right)\;ds\;, \end{array}$$ (24)

being $r_{e}^{A}\left(t\right)$ (resp. $r_{e}^{0}\left(t\right)$) the first component of the periodic solution for the perturbed (resp. unperturbed) system.

Notice that if $\Delta \bar{\alpha }>1$, the external oscillatory input increases the response of the excitatory receiving population. However, the effects of the perturbation onto the receiving population might be also described in terms of enhancement of synchronization in the receiving population. To measure so, we compute the quantities Δ*α* measuring the changes in the maximum and Δ*σ* measuring changes in the half-width.

Following [24], we define Δ*α* as the ratio between the maximum of the excitatory activity *r*_*e*_(*t*) for the perturbed case (*A* ≠ 0), $R_{e}^{A}$, and the maximum of the excitatory activity *r*_*e*_(*t*) for the unperturbed case (*A* = 0), $R_{e}^{0}$, that is,

$$\begin{array}{c} \Delta \alpha =\frac{R_{e}^{A}}{R_{e}^{0}}\;. \end{array}$$ (25)

Notice that if Δ*α* > 1, the external oscillatory input increases the maximal response of the excitatory receiving population. Indeed, we may think that changes in the peak height indicate whether the external forcing synchronizes (Δ*α* > 1) or desynchronizes (Δ*α* < 1) the spikes of the target circuit.

The factor Δ*σ* provides the rate change of the half-width of the E-volley due to the external stimulus. It is defined as

$$\begin{array}{c} \Delta \sigma =\frac{HW^{A}/qT}{HW^{0}/T^{*}},\;\text{with}\;HW^{A}=\frac{1}{2}\left(t_{2}^{A}-t_{1}^{A}\right), \end{array}$$ (26)

where $t_{2}^{A}$ and $t_{1}^{A}$ correspond to the two times ($t_{1}^{A}<t_{2}^{A}$) at which the firing rate is equal to half of its maximum ($r_{e}^{A}=\frac{1}{2}\left(r_{e}^{A,\text{max}}+r_{e}^{A,\text{min}}\right)$). Notice that since we will deal with oscillators of different periods, we must normalize the time difference by its corresponding period.

### 4.7 Vector strength

When a distractor of different frequency than the primary input is applied, the time-*T*_1_ map of the solution of the phase Eq (8) does not satisfy the conditions to compute the rotation number, since the second input (the distractor) is not *T*_1_-periodic. In this case, we still regard the phase in intervals of *T*_1_ time units (although they are now time-dependent) in order to analyse whether the distractor disturbs the entrainment and by which amount. To do so, we compute the synchronization index (SI) [38], also known as vector strength or Kuramoto order parameter [39]. It is a measure of how clustered are the events over a cycle. To compute the SI one associates to each event a vector on the unit circle with a phase angle and computes the mean vector. The SI is given by the length of the mean vector. That is,

$$\begin{array}{c} Z=re^{i\phi }=\frac{1}{N}\sum_{j=1}^{N}e^{i\theta_{j}},\;r=\left|Z\right|. \end{array}$$

Notice that perfect clustering is obtained when *r* = 1, whereas if phases are scattered around the circle, then *r* ≈ 0 (see S6 Fig).

### 4.8 Computation of the phase shift of the numerical PRC

The Phase Response Curve in Fig 8D provides the phase shift due to the application of a square wave current to the oscillation emerging in the E-I network (1)-(2) when entrained by two identical inputs in anti-phase. It is computed as follows. For every phase *t*/*T*_0_ ∈ [0, 1) of the oscillator, we apply a square wave current pulse (amplitude 1.5, duration 2 ms) and compute $PRC=\frac{T_{0}-T_{1}}{T_{0}}$, being *T*_1_ the period of the first cycle after the pulse application and *T*_0_ the period of the entrained oscillator.

The plot also shows, as a function of the phase, if the (entrained) oscillator switches between attended stimulus. To detect whether there is a change in the effective input, we perform the following steps:

1. Before applying the pulse (and once having converged to the periodic orbit) we measure, in a single cycle, the time distance from the maximum of the excitatory firing rate, $t_{e}^{bf}$, to the maximum of each input, $t_{p1}^{bf}$ and $t_{p2}^{bf}$. If $t_{p1}^{bf}$ or $t_{p2}^{bf}$ are larger than $t_{e}^{bf}$ we consider the preceding input peak. Finally, we take the difference between these two distances, that is,
  $$\begin{array}{c} d_{\text{bf.pl}}=\left(t_{e}^{bf}-t_{p1}^{bf}\right)-\left(t_{e}^{bf}-t_{p2}^{bf}\right)\;. \end{array}$$ (27)
2. After applying the pulse, we integrate the whole perturbed system for long enough time (we have used 30 periods *T*_0_) so that the transient effects have washed out. Then, we measure again the time distance from the peak of the excitatory firing rate to the peak of each input as in step 1. We take again the difference between these two time distances, keeping the same orientation as in step 1:
  $$\begin{array}{c} d_{\text{af.pl}}=\left(t_{e}^{af}-t_{p1}^{af}\right)-\left(t_{e}^{af}-t_{p2}^{af}\right)\;. \end{array}$$ (28)

A change of sign between *d*_bf.pl_ and *d*_af.pl_ (i.e. sgn(*d*_bf.pl_ ⋅ *d*_af.pl_) = −1) will determine a change in the oscillator’s effective input.

### 4.9 Numerical methods

Equations for the 8-dimensional system (1)-(2) and for the phase Eq (8) were integrated numerically in Matlab using an explicit Runge-Kutta method of order 4–5 (ode45) with an absolute tolerance ranging beween order 10^−12^ and 10^−16^.

Equations of the microscopic model consisting of *N*_*e*_ = *N*_*i*_ = 5000 QIF neurons were integrated using an Euler method with time step Δ*t* = 10^−4^. There is a refractory period for each neuron of duration *T*_*ref*_ = 2 ⋅ *τ*_*e*,*i*_/*V*_*th*_, where *V*_*th*_ = 500 is the voltage threshold and *τ*_*e*,*i*_ = 8 as in [64].

The bifurcation diagram in Fig 2A was computed using the numerical bifurcation analysis toolbox MatCont (a Matlab continuation package) [65, 66].

We used Matlab to analyse and plot the data.

Matlab codes have been released and are available at https://github.com/david-reyner/Matlab-Code.

## Supporting information

S1 Fig Phase-locking regions predicted using the phase reduction show good agreement with actual ones. Boundaries of the phase-locking regions (Arnold tongues) between the PING oscillation in Fig 3A and a von Mises input (15) with *κ* = 2 and varying amplitude factor *A* and period ratio *T*/T*. Arnold tongues are computed using the phase reduction (dashed curves) and by exhaustive computation of periodic orbits of perturbed system (1)-(2) (fulfilling the corresponding p:q relationship with the input) until convergence fails (black curves).
(EPS)

S2 Fig Representative periodic orbits within the 1:1 phase-locking region for coherent inputs. (A) 1:1 phase-locking region (predicted by means of the phase reduction) between the PING oscillation in Fig 3A and a von Mises input (15) with *κ* = 2 and varying amplitude factor *A* and period ratio *T*/T*. (B) Projection onto the (*r*_*e*_, *V*_*e*_)-plane of 3 representative periodic orbits of the perturbed system (1)-(2), corresponding to the crosses in Panel A (dashed curves with same color as the crosses). Solid red line corresponds to the projection of the unperturbed limit cycle. (C-E) Evolution of the firing rate variables *r*_*e*_ (red) and *r*_*i*_ (blue) and the von Mises input *Ap*(*t*) (green dashed line) over a single period *T* for the three representative orbits: (C) left-hand side of the Arnold tongue (blue cross), (D) center of the Arnold tongue (turquoise cross) and (C) right-hand side of the Arnold tongue (green cross). Blue and green circles indicate the maximum of the I-cells firing rate and the input, respectively. The magnitude Δ*τ* has been pointed out with a double-head arrow.
(EPS)

S3 FigEntrainment properties within the 1:2 and 2:1 phase-locking regions.(A) Evolution of the firing rate variables *r*_*e*_ (red) and *r*_*i*_ (blue) and the von Mises perturbation for *κ* = 2 (dashed green) for a representative periodic orbit of the perturbed system (1)-(2) within the 1:2 (left) and 2:1 (right) phase-locking regions. (B-E) Factors describing changes in the E-cell response within these phase-locking regions for orbits calculated along (equidistant) sections *A* = ct of the corresponding Arnold tongues, indicated by the color of the curve (ranging from dark blue, *A* = 0.01, to yellow, *A* = 0.2, with increments of size 0.01). The factors are: (B) Δ*τ*, describing the timing between inhibition and input volleys (normalized by the input period *T*), (C) $\Delta \bar{\alpha }$, describing the rate change in the averaged firing rate, (D) Δ*α*, describing the rate change in the maximum of the (excitatory) firing rate, and (E) Δ*σ*, describing the rate change in E-volley half-width. See Methods. Additionally, the factors Δ*τ*, Δ*α* and Δ*σ* within the 2:1 Arnold tongue are also shown for the oscillator’s second peak (dashed lines).(EPS)

S4 Fig Representative periodic orbits within the 1:2 phase-locking region for coherent inputs. (A) 1:2 phase-locking region (predicted by means of the phase reduction) between the PING oscillation in Fig 3A and a von Mises input (15) with *κ* = 2 and varying amplitude factor *A* and period ratio *T*/T*. (B) Projection onto the (*r*_*e*_, *V*_*e*_)-plane of 3 representative periodic orbits of the perturbed system (1)-(2) corresponding to the crosses in Panel A (dashed curves with same color as the crosses). Solid red line corresponds to the projection of the unperturbed limit cycle. (C-E) Evolution of the firing rate variables *r*_*e*_ (red) and *r*_*i*_ (blue) and the von Mises input *Ap*(*t*) (green dashed line) over two periods *T* for the three representative orbits: (C) left-hand side of the Arnold tongue (blue cross), (D) center of the Arnold tongue (turquoise cross) and (C) right-hand side of the Arnold tongue (green cross). Blue and green circles indicate the maximum of the I-cells firing rate and the preceding input volley, respectively. The magnitude Δ*τ* has been pointed out with a double-head arrow.
(EPS)

S5 Fig Representative periodic orbits within the 2:1 phase-locking region for coherent inputs. (A) 2:1 phase-locking region (predicted by means of the phase reduction) between the PING oscillation in Fig 3A and a von Mises input (15) with *κ* = 2 and varying amplitude factor *A* and period ratio *T*/T*. (B) Projection onto the (*r*_*e*_, *V*_*e*_)-plane of 3 representative periodic orbits of the perturbed system (1)-(2) corresponding to the crosses in Panel A (dashed curves with same color as the crosses). Solid red line corresponds to the projection of the unperturbed limit cycle. (C-E) Evolution of the firing rate variables *r*_*e*_ (red) and *r*_*i*_ (blue) and the von Mises input *Ap*(*t*) (green dashed line) over a single period *T* for the three representative orbits: (C) left-hand side of the Arnold tongue (blue cross), (D) center of the Arnold tongue (turquoise cross) and (C) right-hand side of the Arnold tongue (green cross). Blue and green circles indicate the maximum of the I-cells firing rate and the input, respectively. The magnitudes Δ*τ*_1,2_ have been pointed out with double-head arrows.
(EPS)

S6 Fig Phase distribution along the unit circle and synchronization index. Phases of the (non-autonomous) stroboscopic map (at time *T*_1_) with two periodic inputs (primary and distractor) of von Mises type with coherence *κ*_1_ = 2 and *κ*_2_ = 20 and amplitude factors *A*_1_ = *A*_2_ = 0.1. The period varies: (A) *T*_1_/T* = 0.845 and *T*_2_ = *T*_1_, (B) *T*_1_/T* = 0.93 and *T*_2_ = 1.3*T*_1_ and (C) *T*_1_/T* = 1 and *T*_2_ = *T*_1_. (Left column) Distribution of the first 1000 phases along the unit circle and (Right column) plot of the same phases (within the range [0, 2*π*]) as a function of the iterates. The values of the synchronization index for each case are: (A) 0.9850, (B) 0.9234 and (C) 0.0224 (see Fig 6). Three different outcomes are observed in the way the phases are distributed: (A) the iterates converge to a single phase value, (B) the iterates jump from one phase to another within a set of different phase values and (C) the phases fill densely the circle.
(EPS)

S7 Fig Simulations of the mean field model receiving a primary input and a distractor. Firing rate *r*_*e*_ (red) and inhibitory synaptic variable *S*_*ei*_ (green) of the mean field model (1)-(2) before and after applying a distractor to the entrained oscillator by the primary input. The primary input of von Mises type (solid black line) oscillates with a relative frequency of *T*_1_/T* = 0.845 (left column), *T*_1_/T* = 0.93 (middle column) and *T*_1_/T* = 1 (right column) with respect to the unperturbed oscillator and it comes in the form of coherent pulses (*κ*_1_ = 2). The distractor is chosen to be as coherent as the first input (*κ*_2_ = 2). The frequency relationship between the primary stimulus and the distractor is (A) *T*_2_/*T*_1_ = 0.5, (B) 0.75, (C) 1, (D) 1.2 and (E) 1.4. The parameters have been chosen as in Fig 6 so that the plots herein illustrate the corresponding predictions. The simulations consist in the integration of system (1)-(2) with the primary stimulus for 25*T*_1_ cycles (only the last cycle is displayed). After that, the system is additionally perturbed with a distractor for time 10 max(*T*_1_, *T*_2_) (only the first 5 cycles are shown).
(EPS)

S8 Fig Simulations of the full spiking QIF model receiving a primary input and a distractor. Both inputs are of von Mises type with the same coherence (*κ*_1_ = *κ*_2_ = 2) and relative frequency *T*_2_/*T*_1_ = 0.875. The parameters for the primary have been chosen within the 1:1 Arnold tongue as in Fig 6: *A*_1_ = 0.1 and (A) *T*_1_/T* = 0.845 (left-hand side of the Arnold tongue), (B) *T*_1_/T* = 0.93 (middle of the Arnold tongue) and (C) *T*_1_/T* = 1 (right-hand side of the Arnold tongue). For each plot we show the von Mises inputs and the raster plots before (left column) and after (right column) applying the distractor at time 600 ms (each panel shows 200 ms of integration time).
(EPS)

S1 Appendix Mutual information between stimulus state (on/off) and the firing rate of the E-cells. (PDF)
