# Supplementary material for: Phase-locking patterns underlying effective communication in exact firing rate models of neural networks
Source: PLoS Comput Biol. 2022 May 18;18(5):e1009342. doi: 10.1371/journal.pcbi.1009342 (PMC9154197; doi:10.1371/journal.pcbi.1009342)
Supplement: S1 Appendix — (PDF) [file pcbi.1009342.s009.pdf]

Mutual information

We present some preliminary results on how the predictions obtained from the deterministic system extend to systems with noise using tools from information theory.

We have added a zero mean white noise current with a standard deviation A_n to the input and we have compared the firing rate of the E-cells of the target network in the presence of the periodic input with the firing rate in the absence of input for different input frequencies. The differential equations were integrated using the Euler-Maruyama method for stochastic differential equations with time step $\Delta t = 0.005$ ms.

The stimulus states were reduced to 2 and were encoded in the amplitude of a periodic input: $A_1 = 0$ (stimulus off), $A_1 = 0.1$ (stimulus on). The distribution was assumed to be uniform over the range of A_1 considered. Thus, one variable (S) was the stimulus state (off/on) controled by the parameter A_1 ($A_1 = 0, 0.1$) and the other one R was the response of the network. We considered 3 different possibilities for the variable R: the maximum firing rate of the E-cells in each cycle discretized in bins of size 0.01, the half-width of the E-volley in each cycle discretized in bins of size 0.1, and the time average firing rate of the E-cells over one cycle discretized in bins of size 0.25. Thus, mutual information between S and R is computed using the formula

$$I(R,S) = \sum_i \sum_j p(s_i) p(r_j|s_i) \log_2 \frac{p(r_j|s_i)}{p(r_i)},$$

where $I(R;S)$ is the mutual information between the response R and stimulus S; $p(s_i)$ is the probability of presenting stimulus s_i, $P(r_j)$ is the probability of observing response r_j across all trials in response to any stimulus and $p(r_j|s_i)$ is the probability of observing response r_j in response to a stimulus s_i. The latter probabilities are estimated from the histogram of maximum firing rates in bins of size 0.01 obtained from simulations with 500 input cycles per each stimulus. We have repeated the computations with 1000 cycles and the results do not change significantly.

In Figure S9 we show the mutual information between the stimulus state (on or off) described by the amplitude of the input signal ($A_1 = 0, 0.1$) and the maximum firing rate of the E-volley, for different frequencies of the input and noise strength A_n. We also computed mutual information between the stimulus state and the E-volley half-width and the average firing rate of the E-cells over a cycle of the input. Results show that the E-cells encoded the stimulus state (on or off) very well by means of the spike synchronization within the E-population (determined by the maximum and half-width of the E-volley) if the information is encoded in a periodic input signal with higher frequency than the natural gamma cycle, but the communication degrades as the frequency becomes similar to the natural gamma cycle. However, the transmission of information in the average firing of the E-I network is weak at all frequencies.

In Figure S10A we show the mutual information between the primary stimulus (on/off) described by the amplitude of the primary input $A_1 = 0, 0.1$ with frequency $T/T^* = 0.845$ and the firing rate of the E-population in the presence of a distractor of strength $A_2 = 0.1$ and $T_2/T_1 = 1.2$ and white noise. Notice that the mutual

information with the maximum firing rate and half-width remains high when we add the distractor (compare values in Figure S10A with Figure S9D for the frequency $T/T^* = 0.845$). In Figure S10B we present the mutual information between the amplitude of the distractor $A_2 = 0, 0.1$ and the firing rate of the E-cells when the primary input is present $A_1 = 0.1$. We obtain low values compared with the primary.

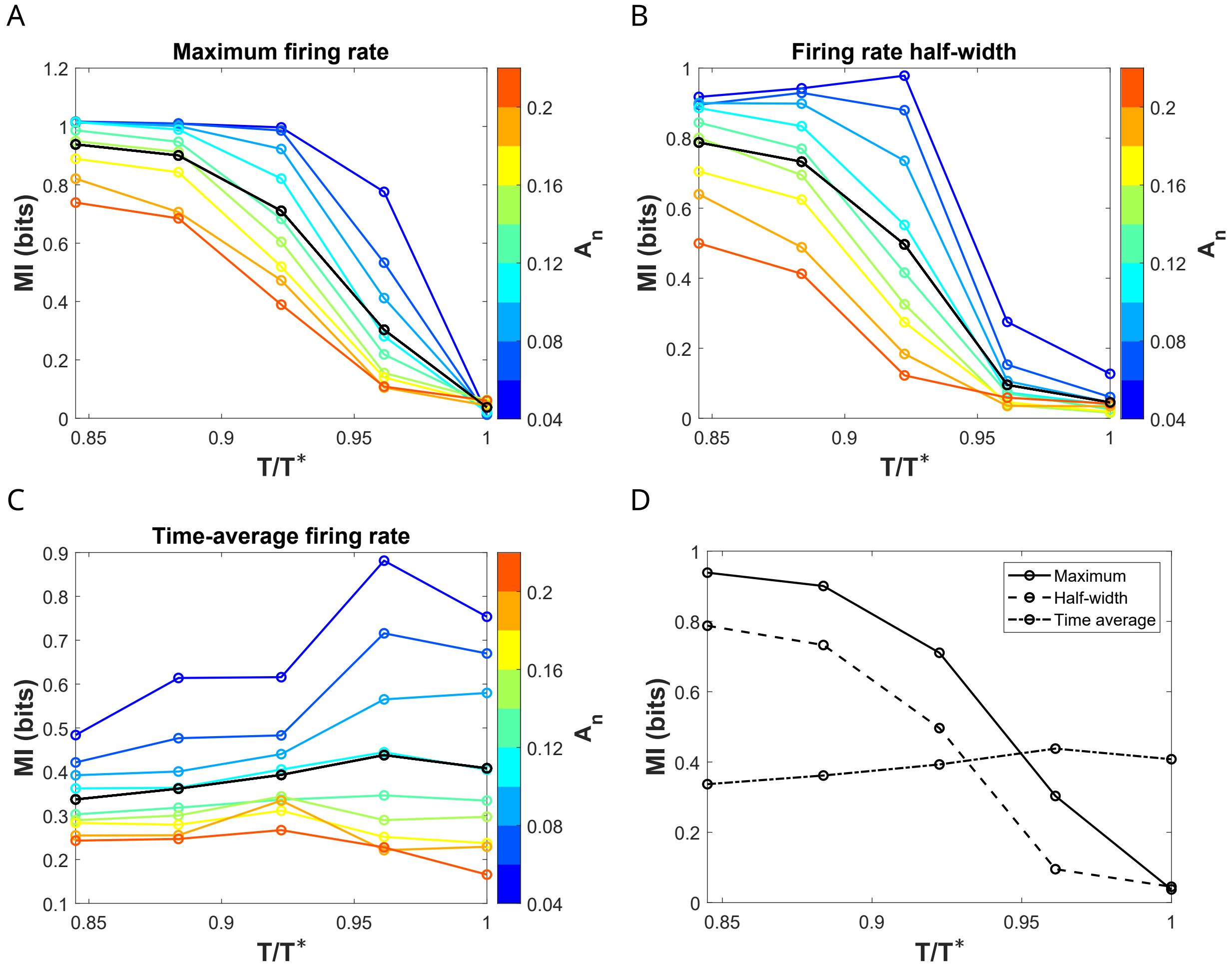

Figure S9: **Mutual information between stimulus state (on/off) and the firing rate of the E-cells is higher for higher input frequencies.** Mutual information between the stimulus state (on/off) corresponding to $A = 0$ (off) and $A = 0.1$ (on) in the input signal (15) and (A) the maximum firing rate of the E-cells over a cycle, (B) the half-width of the E-volleys and (C) the time-average firing rate of the E-cells over a cycle, as a function of the input frequency T/T^* for different values of the noise strength A_n (colorbar). Black curve corresponds to the average over the different noise values. (D) Averaged curves from panels (A-C).

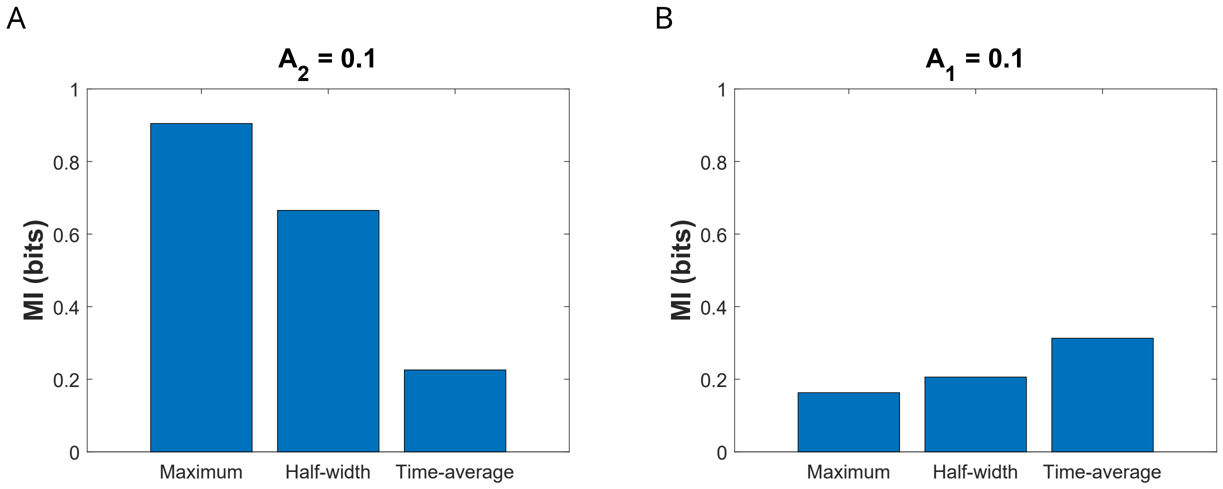

Figure S10: **Mutual information between stimulus state (on/off) and the firing rate of the E-cells is maintained in the presence of a distractor for stimuli encoded in high frequency inputs.** Mutual information between the target stimulus state (on/off) corresponding to strength $A_1 = 0$ (off) and $A_1 = 0.1$ (on) in the von Mises input with frequency $T_1/T^* = 0.845$ and the maximum firing rate of the E-cells over a cycle, the half-width of the E-volleys and the time-average firing rate of the E-cells over a cycle in the presence of a distractor of von Mises type with $A_2 = 0.1$ and $T_2/T_1 = 1.2$ and noise of strength $A_n = 0.1$. (B) Mutual information between the amplitude of the distractor $A_2 = 0, 0.1$ and the same firing rate measures as in panel A when the primary has an amplitude fixed at $A_1 = 0.1$.
